# Four cellulose-active lytic polysaccharide monooxygenases from *Cellulomonas* species

**DOI:** 10.1186/s13068-020-01860-3

**Published:** 2021-01-23

**Authors:** James Li, Laleh Solhi, Ethan D. Goddard-Borger, Yann Mathieu, Warren W. Wakarchuk, Stephen G. Withers, Harry Brumer

**Affiliations:** 1 Michael Smith Laboratories, University of British Columbia, 2185 East Mall, Vancouver, BC V6T 1Z4 Canada; 2 Department of Biochemistry and Molecular Biology, University of British Columbia, 2350 Health Sciences Mall, Vancouver, BC V6T 1Z3 Canada; 3 Department of Chemistry, University of British Columbia, 2036 Main Mall, Vancouver, BC V6T 1Z1 Canada; 4 Department of Biological Sciences, University of Alberta, Edmonton, AB T6G 2E9 Canada; 5 Department of Botany, University of British Columbia, 3200 University Blvd, Vancouver, BC V6T 1Z4 Canada; 6 BioProducts Institute, University of British Columbia, 2385 East Mall, Vancouver, BC V6T 1Z4 Canada

**Keywords:** LPMO, AA10, Cellulose, Biomass, Bioethanol, Bioproducts

## Abstract

**Background:**

The discovery of lytic polysaccharide monooxygenases (LPMOs) has fundamentally changed our understanding of microbial lignocellulose degradation. *Cellulomonas* bacteria have a rich history of study due to their ability to degrade recalcitrant cellulose, yet little is known about the predicted LPMOs that they encode from Auxiliary Activity Family 10 (AA10).

**Results:**

Here, we present the comprehensive biochemical characterization of three AA10 LPMOs from *Cellulomonas flavigena* (*Cfla*LPMO10A, *Cfla*LPMO10B, and *Cfla*LPMO10C) and one LPMO from *Cellulomonas fimi* (*Cfi*LPMO10)*.* We demonstrate that these four enzymes oxidize insoluble cellulose with C1 regioselectivity and show a preference for substrates with high surface area. In addition, *Cfla*LPMO10B, *Cfla*LPMO10C, and *Cfi*LPMO10 exhibit limited capacity to perform mixed C1/C4 regioselective oxidative cleavage. Thermostability analysis indicates that these LPMOs can refold spontaneously following denaturation dependent on the presence of copper coordination. Scanning and transmission electron microscopy revealed substrate-specific surface and structural morphological changes following LPMO action on Avicel and phosphoric acid-swollen cellulose (PASC). Further, we demonstrate that the LPMOs encoded by *Cellulomonas flavigena* exhibit synergy in cellulose degradation, which is due in part to decreased autoinactivation.

**Conclusions:**

Together, these results advance understanding of the cellulose utilization machinery of historically important *Cellulomonas* species beyond hydrolytic enzymes to include lytic cleavage. This work also contributes to the broader mapping of enzyme activity in Auxiliary Activity Family 10 and provides new biocatalysts for potential applications in biomass modification.

## Background

The development of second-generation bioethanol and other chemicals from renewable plant biomass is primarily limited by the intrinsic difficulty in depolymerizing the complex matrix of cellulose, hemicellulose, and lignin comprising lignocellulose. Specifically, the highly stabilizing hydrogen bonding network and stacking interactions between cellulose chains [1, 2], together with the composite nature of lignocellulose, limit the ability of hydrolytic enzymes to access the scissile β(1,4)-glucan linkages. As such, many novel methodologies for biomass pretreatment have been developed over the past decades [3, 4] to overcome the problem of substrate recalcitrance. In harness, enzyme discovery and enzyme engineering approaches have continued to deliver new and improved biocatalysts for lignocellulose degradation. Indeed, bacteria and fungi have evolved elegant strategies for complete biomass breakdown, which has prompted detailed exploration of the secretomes of certain highly cellulolytic organisms [5–8]. Traditionally, much of the research on microbial lignocellulose breakdown has focused on the glycoside hydrolases, and specifically the secreted endo-glucanases (EG) and exo-glucanases (cellobiohydrolases, CBH) responsible for carbohydrate saccharification [9–15]. As a result, many cellulolytic enzyme cocktails [16, 17] and engineered organisms [18–22] have been developed, which have significantly advanced industrial biomass conversion.

Nonetheless, it has been long recognized that hydrolytic cellulases are not particularly efficient in catalyzing the conversion of paracrystalline cellulose into fermentable glucose. Seminal studies over the last decade have revealed the existence of copper-dependent lytic polysaccharide monooxygenases (LPMOs), which oxidatively cleave the cellulose chain thereby potentiating the activity of hydrolytic enzymes (chitin-, starch-, and hemicellulose-active LPMOs are also known) [23–29]. Several sequence-similar families of LPMOs are now known, which are classified in the CAZy database as Auxiliary Activity (AA) families AA9, AA10, AA11, AA13, AA14, AA15, and AA16 [30–34]. LPMOs are currently known to be encoded in genomes across all kingdoms of life, as well as viruses [35], and catalyze cleavage of a wide diversity of substrates. In addition to boosting the activity of hydrolytic enzymes for nutrient acquisition, LPMOs have also recently been implicated in other biological roles, including plant defense [36] and microbial copper acquisition and virulence [37].

*Cellulomonas* bacteria have historically been key model systems for cellulase research. *Cellulomonas fimi* in particular has attracted research interest since the late 1960s, when it was first shown to be capable of completely saccharifying pretreated sugarcane bagasse [38]. Subsequently, the cellulases and hemicellulases of *C. fimi* have been the subject of early gene cloning, recombinant expression, and detailed mechanistic and structural studies, which have significantly informed the broader field of glycosidase and carbohydrate-binding module research [9–14, 39–65]. Despite this rich history, whole-genome sequences of *C. fimi* and *C. flavigena* only became available in the past decade [66, 67], revealing that these species encode large and diverse collections of glycoside hydrolases (see http://www.cazy.org/b1637.html and http://www.cazy.org/b1235.html for current censuses) [68]. A recent proteomic analysis [5] has subsequently identified key components of the *C. fimi* and *C. flavigena* secretomes, and further indicated that these bacteria encode one and four predicted LPMOs from AA10, respectively, which were previously uncharacterized. Genomic analysis [66, 67] indicates that all of the *C. flavigena* AA10-encoding genes are surrounded by diverse, unrelated CAZyme-encoding genes, while the sole AA10-encoding gene from *C. fimi* is not proximal to any known CAZyme-encoding genes.

Here, we describe the comprehensive biochemical characterization of four cellulolytic AA10 LPMOs from *C. fimi* and *C. flavigena*: *Cfi*LPMO10, *Cfla*LPMO10A, *Cfla*LPMO10B, and *Cfla*LPMO10C (nomenclature according to ref. [58]). We demonstrate the substrate specificity and regioselectivity of each LPMO using enzyme kinetic and product analysis by HPLC and mass spectrometry. The thermostability of each LPMO was investigated and evidence is presented concerning the critical role that copper ligand binding plays in enabling refolding following thermal denaturation. In addition, we examined the morphological effects of LPMO activity on cellulose surfaces using transmission and scanning electron microscopy. Finally, through activity progress curves, we demonstrate that the distinct LPMOs encoded by *C. flavigena* play synergistic roles in cellulose breakdown.

## Results

### Primary sequence analysis of *Cellulomonas* catalytic modules

All *Cellulomonas* LPMOs are multi-modular proteins containing a SEC pathway secretion signal peptide, a catalytic AA10 module, and a C-terminal CBM2 that is indicative of cellulose or chitin binding [45, 69–71]. Pairwise sequence alignment of the AA10 modules indicated that the single *C. fimi* ortholog, *Cfi*LPMO10, was most similar to *Cfla*LPMO10B (74% sequence identity, 80% sequence similarity, Additional file 3: Table S1). Among the *C. flavigena* LPMOs, *Cfla*LPMO10B and *Cfla*LPMO10C were the most similar to each other (60% pairwise identity, 71% similarity). Notably, *Cfla*LPMO10D, encoded by Cfla_0490, was the most distinct sequence, sharing ca. 30% pairwise similarity and 20% pairwise identity with all of the other *Cellulomonas* AA10 members.

**Fig. 1:**
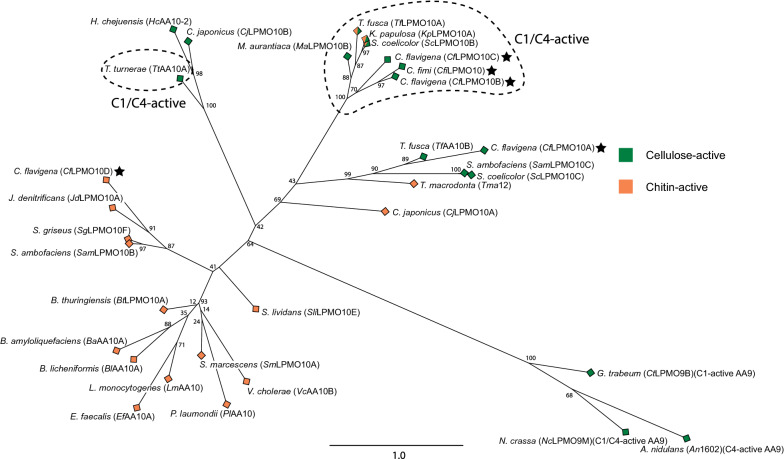
Maximum-likelihood phylogenetic tree of all currently characterized AA10 LPMO catalytic modules. Full-length protein sequences were retrieved from GenBank via the CAZy database and manually truncated to remove the signal peptide and C-terminal domains (guided in part by SignalP and BLASTP analysis). The catalytic sequences were then aligned using MUSCLE and manually refined to preserve the position of the catalytic residues prior to maximum-likelihood analysis using RAxML 8.2.10 utilizing a JTT matrix-based nucleotide substitution model and automatic halting bootstraps (702 bootstraps). Three characterized AA9 LPMO catalytic module sequences with different regioselective activities on chitin were used as outgroups (*Gt*LPMO9B [131], AN1602 [132], and *Nc*LPMO9D [133])**.** The input and output files are provided as Additional file1: S1 and Additional file2: S2

Multiple sequence analysis and Maximum-Likelihood phylogenetic analysis with all characterized AA10 LPMOs presently listed in the CAZy database revealed the likely substrate specificities of each *Cellulomonas* AA10 member. *Cfi*LPMO10, *Cfla*LPMO10A, *Cfla*LPMO10B, and *Cfla*LPMO10C segregated into a predominantly cellulose-specific clade, whereas *Cfla*LPMO10D clustered within an exclusively chitin-active clade (Fig. 1). These clades correspond to Clade I and Clade II, respectively, as described by Book et al. [72]. In addition, *Cfla*LPMO10A further segregated into a subclade of C1-regiospecific cellulose-active LPMOs, including *Thermobifida fusca* LPMO10B [73], *Streptomyces coelicolor* LPMO10C [74], and *Streptomyces ambofaciens* LPMO10C [75]. On the other hand, *Cfi*LPMO10, *Cfla*LPMO10B, and *Cfla*LPMO10C segregated into a subclade comprising cellulose-active LPMOs possessing both C1-hydroxylating and C4-dehydrogenating activity, including *Thermobifida fusca* LPMO10A [73], *Streptomyces coelicolor* LPMO10B [74], and *Micromonospora aurantiaca* LPMO10B [76]. All copper-binding LPMOs contain a conserved histidine brace motif [77–79] presented on the substrate-interacting face of an Immunoglobulin-like/Fibronectin III-like beta-sandwich fold comprising 7–9 beta strands [31, 32, 80].

**Fig. 2:**
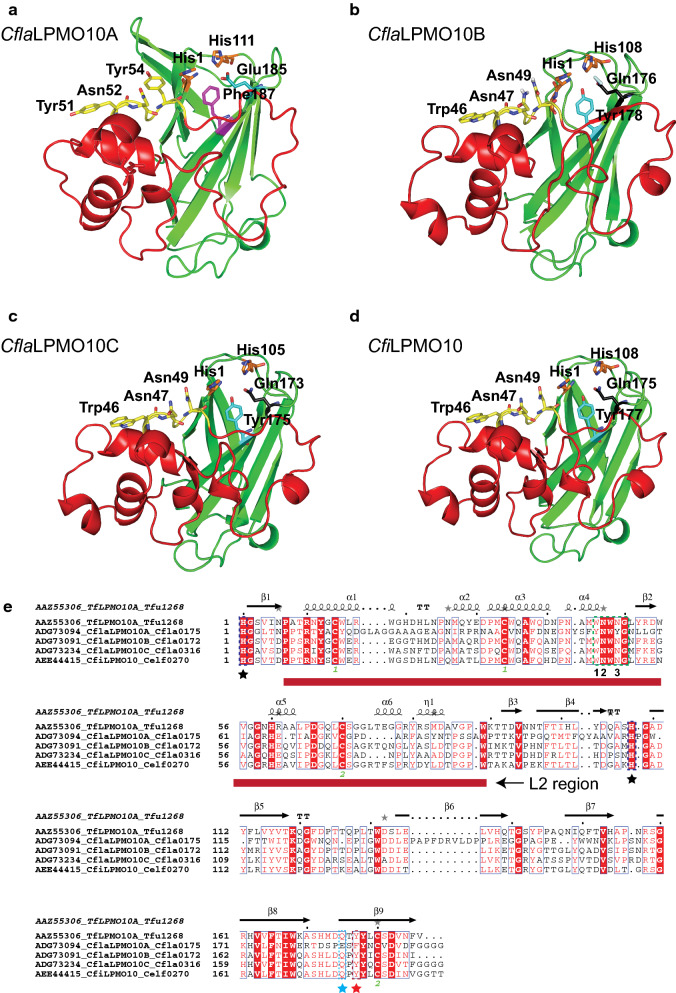
Homology models and primary sequence alignment of *Cellulomonas* LPMO catalytic modules. **a**–**d** Cartoon representation of three-dimensional homology models of *Cfla*LPMO10A, *Cfla*LPMO10B, *Cfla*LPMO10C, and *Cfi*LPMO10, respectively, generated using the Phyre^2^ server with *T. fusca* LPMO10A as the template structure [81]. The active-site histidine residues are depicted as orange sticks, the three conserved residues of the cellulose-active motif are depicted as yellow sticks, the axial tyrosine or phenylalanine residues are depicted as turquoise or pink sticks, respectively, and the catalytic glutamate or glutamine residues are depicted as teal sticks. Residue numbering corresponds to His-1 in the mature protein sequence. The L2 region is colored red to distinguish it from the immunoglobulin-like β-sandwich core colored in green. **e** Sequence and secondary structure alignment of *Cellulomonas* LPMOs compared to *T. fusca *LPMO10A [73]. The cellulose-active motif is indicated by a green dotted box and the positioning of the conserved residues mentioned in-text are numbered 1–3. The two active site histidine residues are indicated with black star symbols. The red dotted box with star indicates the position of the axial aromatic residue. The position of the catalytic glutamate or glutamine is indicated with a teal star

The Phyre^2^ server [81] was used to generate tertiary structure homology models of the four *Cellulomonas* LPMO catalytic modules, which clearly revealed the conserved beta-sandwich fold (Fig. 2 and Additional file 3: Figure S2). The sequence between strands β1 and β3 of AA10 LPMOs (β1 and β2 in AA9s) contains a highly variable region of loops and helices, termed the L2 region, which has been shown to be important in substrate binding and recognition [23, 80, 82, 83]. *Cfi*LPMO10, *Cfla*LPMO10A, *Cfla*LPMO10B, and *Cfla*LPMO10C all contained a long L2 region comprising a cellulose-binding signature motif [23, 84] (Fig. 2e). In contrast, *Cfla*LPMO10D contained a slightly shorter L2 region with a distinct chitin-binding signature motif [23, 84] (Additional file 3: Figure S2). Within these signature motifs, the first, second, and fourth residues have been shown to be critical in substrate recognition and regioselectivity [29, 85–88] (Fig. 2e). The first aromatic residue, predominantly Trp in cellulolytic AA10 members and Tyr in chitinolytic AA10 members, is located on the binding face and was shown through molecular dynamic simulations [85, 87] and NMR [29] to participate in stacking interactions with cognate substrates. The residue directly adjacent is a strictly conserved Asn or Glu in cellulose- and chitin-active AA10 LPMOs, respectively [88]. *Cfi*LPMO10, *Cfla*LPMO10A, *Cfla*LPMO10B, and *Cfla*LPMO10C each contain Asn at this position, whereas *Cfla*LPMO10D contains Glu (Fig. 2). The fourth residue of the specificity signature motif is Phe or Gln in strict C1-regiospecific cellulose- and chitin-active AA10 LPMOs [88], and Asn in mixed C1/C4 cellulose-active AA10 LMPOs [88] which corroborated with our multiple sequence analysis (Additional file 3: Figure S3). *Cfi*LPMO10, *Cfla*LPMO10B, and *Cfla*LPMO10C each contain Asn at this position (Fig. 2b–d), while *Cfla*LPMO10D contains Gln (Additional file 3: Figure S2A). Notably, *Cfla*LPMO10A contains a Tyr at this position (Additional file 3: Fig. 2a), which may be structurally and functionally homologous to Phe. In addition, only *Cfla*LPMO10A was found to contain a Glu at position 185, which was shown to be highly conserved in strict C1-oxidizing cellulose-active AA10 enzymes [78, 89], whereas the other LPMOs have a Gln at this position.

Taken together, these bioinformatic analyses suggest that *Cfi*LPMO10, *Cfla*LPMO10A, *Cfla*LPMO10B, and *Cfla*LPMO10C are likely to be cellulose-active, whereas *Cfla*LPMO10D is likely to be chitin-active. Notably, secretomic analysis of *C. fimi* and *C. flavigena* revealed that the production of *Cfi*LPMO10, *Cfla*LPMO10A, *Cfla*LPMO10B, and *Cfla*LPMO10C were all upregulated when grown on carboxymethyl cellulose (CMC) [5]. Hence, we selected the four putative cellulose-active LPMOs for recombinant production and further characterization.

### Recombinant production of *Cellulomonas* LPMOs

The four putative cellulose-active LPMOs (*Cfla*LPMO10A, *Cfla*LPMO10B, *Cfla*LPMO10C, and *Cfi*LPMO10) each contain a secretory signal peptide that is predicted to target these proteins to the SEC pathway [90]. cDNA encoding the AA10 modules, including the native SEC secretion signal peptide, of *Cfi*LPMO10, *Cfla*LPMO10A, *Cfla*LPMO10B, and *Cfla*LPMO10C, was cloned from genomic DNA with the addition of a C-terminal His_6_ purification tag and transformed into *E. coli* for expression. Secretion of LPMO proteins through the SEC pathway in *E. coli* has previously resulted in accumulation of recombinant protein in the periplasm [91, 92] and the culture medium [93, 94], with the former necessitating periplasmic fractionation. We observed that the majority of the recombinant *Cellulomonas* LPMOs were found in the media, with negligible amounts in the periplasm. Thus, all targets were subsequently purified from the cell-free supernatant without the need of mechanical lysis and soluble fraction separation. Following immobilized metal affinity chromatography, production in shake flasks yielded 1–2 mg of soluble *Cfi*LPMO10, *Cfla*LPMO10B, and *Cfla*LPMO10C per liter of culture medium and 5–10 mg of *Cfla*LPMO10A per liter of culture medium. Protein purity was verified by polyacrylamide gel electrophoresis (Additional file 3: Figure S4), and confirmation of the correct processing of the signal peptide to generate the critical N-terminus histidine was confirmed through intact protein mass spectrometry (Additional file 3: Figure S1; *Cfla*LPMO10A residues 1–204, calc. *Mr* 23639.9 Da, obs. *Mr* 22,273 Da, corresponding to a loss of SAAAALEHHHHHH at the C-terminus; *Cfl*LPMO10B residues 1–239, calc. *Mr* 22822.2 Da, obs. *Mr* 22822.0 Da; *Cfl*LPMO10C residues 1–204, calc. *Mr* 22741.9 Da*,* obs. *Mr* 22740.0 Da; and *Cf*LPMO10 residues 1-203, calc. *Mr* 22810.3 Da, obs. *Mr* 22808.0 Da).

### Substrate specificity

**Fig. 3:**
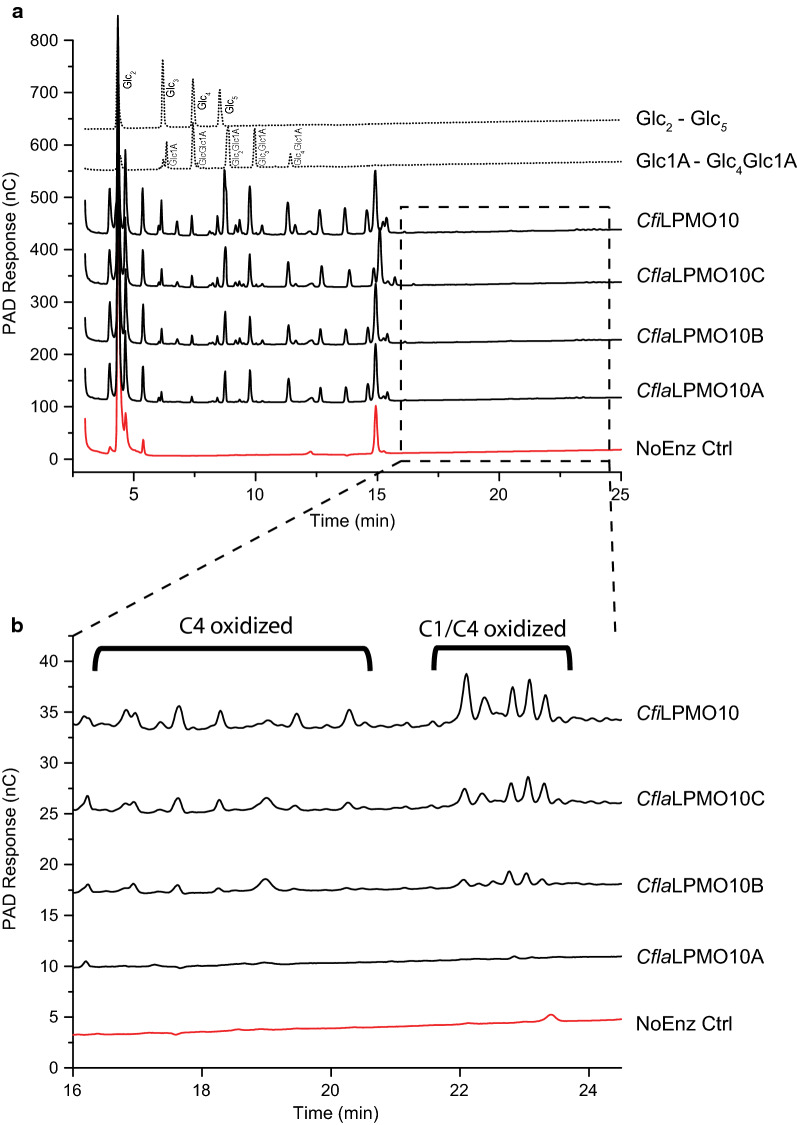
HPAEC-PAD analysis of *Cellulomonas* LPMO activity on PASC. **a** Chromatogram over the retention times 3–25 min **b** Expansion of the 16–24 min region. Native and C1-oxidized cello-oligosaccharide standard peaks are annotated. All activity assays were conducted using 1 μM of enzyme, 0.1% PASC, and 1 mM ascorbic acid over 16 h at 37 °C. The negative control (NoEnz Ctrl) contained all assay components except enzyme

The canonical substrates of LPMOs are insoluble cellulose and chitin, although some LPMOs have been shown to be active on soluble hemicelluloses and starch [23]. Thus, the activities of the purified *Cellulomonas* LPMOs were initially screened against a large panel of polysaccharide and oligosaccharide substrates in an overnight (16 h) end-point assay under aerobic conditions using ascorbic acid as the reductant. *Cfi*LPMO10, *Cfla*LPMO10A, *Cfla*LPMO10B, and *Cfla*LPMO10C were all active on insoluble cellulosic substrates, including microcrystalline cellulose (Avicel), phosphoric acid-swollen cellulose (PASC), bacterial cellulose (BC) from *Komagataeibacter xylinus* (also known as *Acetobacter xylinum* or *Gluconacetobacter xylinus*), Northern Bleached Softwood Kraft Pulp (NBSKP), as well as cellulose nanocrystals (CNC) and cellulose nanofibrils (CNF) derived from NBSKP. Representative product profiles from enzyme incubations with PASC determined by HPLC are shown in Fig. 3. No activity was observed on chitin, chitosan, tamarind xyloglucan, barley mixed-linkage β-glucan, carboxymethyl cellulose, hydroxyethyl cellulose, beechwood xylan, curdlan, starch, glucose, and soluble cello-oligosaccharides (Glc_2_ through Glc_6_).

**Fig. 4:**
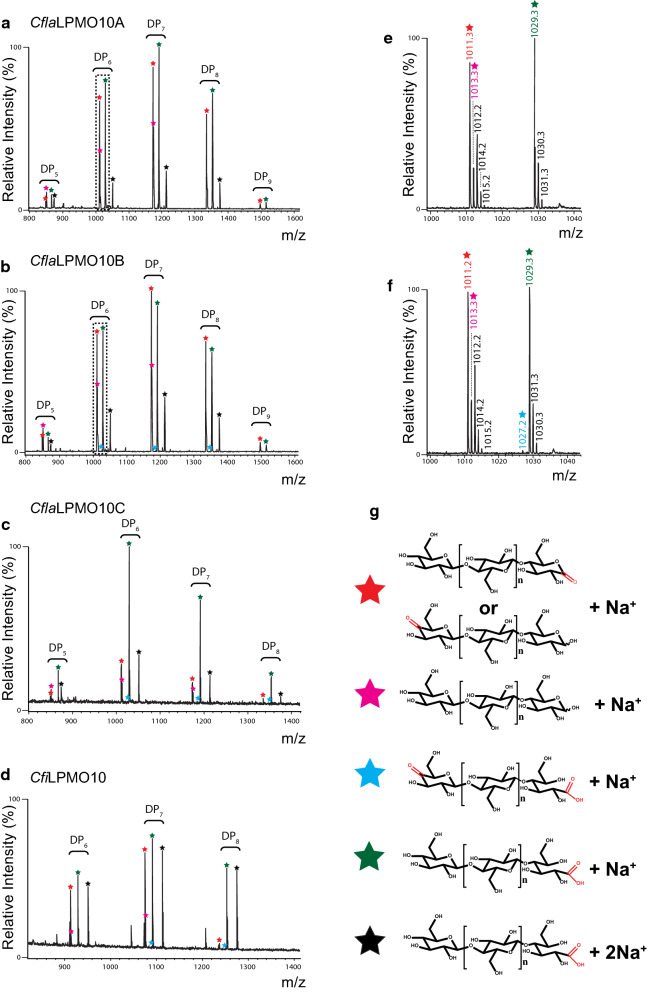
MALDI-TOF product analysis of *Cellulomonas* LPMO activity on PASC. **a**–**d** Mass spectrum of *m/z* 800–1600 showing native and oxidized cello-oligosaccharide products between DP_5_—DP_8_ [M + Na^+^] released by the *Cellulomonas* LPMO indicated above each respective panel. **e**, **f** Representative expanded mass spectra of *m/z* 1000–1040 depicting DP_6_ [M + Na^+^] peaks from *Cfla*LPMO10A and *Cfla*LPMO10B corresponding to native, C1-, and mixed C1/C4-oxidized cello-oligosaccharides. **g** Symbols used to denote native and oxidized cello-oligosaccharide products corresponding to peaks in mass spectra. Red stars are monosodiated aldonic and lactone forms of C1-oxidized cello-oligosaccharides. Pink stars are monosodiated native cello-oligosaccharides; teal stars are monosodiated doubly oxidized C1 aldonic acid/C4 ketoaldose oligosaccharides; green stars are monosodiated aldonic acid forms of C1-oxidized cello-oligosaccharides; and black stars are disodiated aldonic acid forms of C1-oxidized cello-oligosaccharides

Initial analysis of the product profiles by HPLC of the action of the four *Cellulomonas* LPMOs on cellulosic substrates, as exemplified by PASC, clearly showed the production of C1-oxidized cello-oligosaccharides (Fig. 3a), for which standard samples were readily produced. By analogy with previous studies [74, 76, 95], the presence of peaks of longer retention time (> 16 min), suggested the possibility of additional C4 and mixed C1/C4-oxidized products (Fig. 3b) in the assay supernatant of *Cfi*LPMO10, *Cfla*LPMO10B, and *Cfla*LPMO10C. To confirm the ability of these *Cellulomonas* LPMOs to catalyze C4 oxidation, the released soluble products were analyzed by MALDI-TOF MS. Consistent with the HPLC data, the mass spectrum evidenced products with *m/z* values corresponding to [M + Na^+^] adducts of Glc_5_–Glc_8_ cello-oligosaccharides and their C1-oxidized counterparts (Fig. 4). Specifically, pseudomolecular ions with + 16 m*/z* and -2 m*/z* were also observed, which were consistent with the aldonic and lactone forms of C1-oxidized cello-oligosaccharides (Fig. 4e, f). Mass spectra of the products of *Cfi*LPMO10, *Cfla*LPMO10B, and *Cfla*LPMO10C also evidenced products with pseudomolecular ions of -4 m*/z*, which corresponds to doubly oxidized C1 aldonic acid/C4 ketoaldose oligosaccharides (Fig. 4e); C4 ketoaldose oligosaccharides are isobaric with C1-oxidized lactones and are thus indistinguishable by MS. Together, the HLPC and MS data demonstrate that all four *Cellulomonas* LPMOs predominantly catalyze C1 regioselective oxidation of cellulose. Whereas *Cfla*LPMO10A appears to be C1 regiospecific, *Cfi*LPMO10, *Cfla*LPMO10B, and *Cfla*LPMO10C are also capable of C4 oxidation. These results are consistent with the regioselectivity predicted in the phylogenetic analysis.

**Fig. 5:**
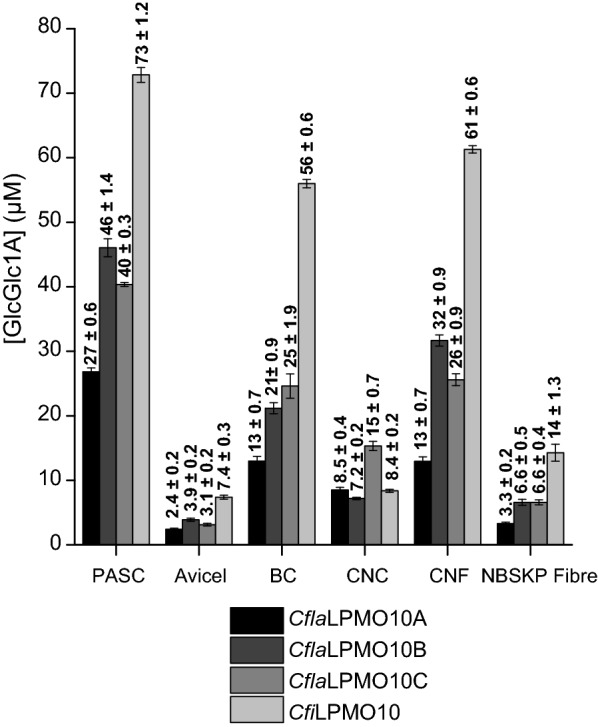
C1-oxidized ends produced by 1 μM *Cellulomonas* LPMOs after 16 h on a panel of insoluble cellulose substrates. All substrates were assayed at 0.1% except NBSKP fiber which was assayed at 1% (no activity was detected at 0.1% NBSKP fiber). For each substrate, *T. reesei* cellulase cocktail (Celluclast; Sigma-Aldrich, P/N: C2730-30) was used to convert all C1-oxidized products into cellobionic acid and quantified as the total C1-oxidized ends generated (μM). Total oxidized ends were obtained by quantifying cellobionic acid by HPLC following hydrolysis of soluble products with *T. reesei* Celluclast enzyme cocktail. Each time point represents the average of three independent assays measured singly by HPLC, with error bars indicating the standard error of the mean

To simplify quantitation by HPLC, soluble C1-oxidized products were hydrolyzed using a cellulolytic cocktail to glucose, cellobiose, and cellobionic acid. The peak corresponding to cellobionic acid was then used to approximate *Cellulomonas* LPMOs activity after 16 h incubations with Avicel, PASC, BC, NBSKP, CNC, and CNF. Avicel and PASC, in particular, represent commonly used model cellulose substrates of differing morphologies: Avicel, which is produced by acid hydrolysis of wood pulp, is a representative Cellulose I (parallel microfibrillar) substrate with a low surface area and high crystallinity index (ca. 60% [96]). PASC is produced by phosphoric acid treatment of Avicel, which yields Cellulose II (antiparallel microfibrils) with a high surface area to volume ratio and a relatively low degree of crystallinity (ca. 30% [97]) [95, 96, 98–100]. As shown in Fig. 5, all four *Cellulomonas* LPMOs are most active on PASC (30—70 μM of oxidized products after 16 h). For all of the LPMOs, substrates with higher degrees of crystallinity and/or lower surface area, e.g., Avicel and CNC, were the poorest substrates. BC and CNF were intermediate as substrates, likely due to higher surface areas; BC is extruded as fine ribbons [101] and CNF is mechanically fibrillated from NBSKP. Interestingly, industrially relevant wood pulp fibers (NBSKP) were the poorest substrates for the LPMOs, and activity was only detected at very high substrate loadings (1% NBSKP vs 0.1% for all other substrates). CfiLPMO10 exhibited the highest activity of all four LPMOs across the entire range of cellulose substrates, while the three *C. flavigena* homologs exhibited comparable activities to each other.

Both oxygenase (O_2_ cosubstrate) [102, 103] and peroxygenase (H_2_O_2_ cosubstrate) [104] activity have been reported for individual LPMOs. Hence, each *Cellulomonas* LPMO was assayed for the ability to utilize hydrogen peroxide as a cosubstrate in both aerobic and anaerobic conditions. *Cfla*LPMO10A was the only enzyme for which peroxygenase activity was measurable (Additional file 3: Figure S5). Under anaerobic conditions (i.e., with 100 μM H_2_O_2_ as the sole cosubstrate and 1 mM ascorbic acid as the reductant), a rapid burst of activity in the first minute of reaction was observed; however, this activity quickly ceased at 10 min. Under aerobic conditions, the addition of H_2_O_2_ likewise resulted in an initial increase in the rate of cellobionic acid production, but again rapid inactivation was observed, suggesting that *Cfla*LPMO10A succumbs to oxidative damage more rapidly in the presence of peroxide. In light of these results, all further quantitative LPMO assays were performed aerobically, with ambient molecular oxygen as the sole cosubstrate.

### pH Dependence and thermostability

All four cellulose-active *Cellulomonas* LPMOs remained catalytically active between pH 4.5 and 10.5 and were generally difficult to inactivate. Indeed, as exemplified by *Cfla*LPMO10A, activity was generally observed to be undiminished after boiling for 30 min or after addition of high concentrations of ammonium hydroxide (0.5 M) at room temperature. Only the addition of EDTA at room temperature led to a loss of activity (Additional file 3: Figure S6). To investigate the basis of this high temperature and pH stability, a Thermofluor analysis [105, 106] was used to assess the melting temperature of *Cellulomonas* LPMOs at various pH conditions. Each *Cellulomonas* LPMO was shown to unfold at around 45–55 °C in the storage buffer 20 mM Tris–HCl, pH 8. For all four enzymes, it was generally observed that the highest melting temperatures were at neutral to alkaline pH, with a reduction of melting temperatures below pH 5 (Additional file 3: Figure S7A-7D). Thermofluor analysis also indicated that all four *Cellulomonas* LPMOs were able to refold following cooling at room temperature, as evidenced by *T*_m_ values from repeat melting curves. Subsequently, differential scanning calorimetry (DSC) of *Cfla*LPMO10A at various pH conditions confirmed that the protein melting temperature was essentially unchanged at neutral to alkaline pH following repeated temperature cycling (Additional file 3: Figure S8).

The ability to regenerate active enzymes by refolding was confirmed by assaying each LPMO for the production of oxidized Glc_4_–Glc_6_ oligosaccharides before and after boiling. (*Cfla*LPMO10A was fully active following cooling, whereas *Cfla*LPMO10B, *Cfla*LPMO10C, and *Cfi*LPMO10 retained ca. 70–80% of their initial activity (Additional file 3: Table S2). The apparent inability of the LPMOs to refold at low pH is likely due to protonation of active-site histidines, which would interfere with copper coordination. Indeed, when copper was chelated with D-penicillamine (DPA) in the Thermofluor analysis, we were unable to observe a second melting curve (Additional file 3: Figure S7E–G) following cooling to allow spontaneous refolding with any of the LPMOs at pH 8. Notably, the presence of DPA did not significantly affect the melting temperature of LPMOs during the first heating cycle, suggesting that copper was not likely to have been extracted from the holoenzymes. Taken together these results clearly demonstrate the critical effect of bound copper ligand on the thermal stability of LPMOs.

### Effect of cellulose morphology on LPMO activity and stability

After establishing the cellulose specificity and regioselectivity of the four *Cellulomonas* LPMOs, we sought further insight into the effect of substrate morphology on enzyme activity and stability using Avicel and PASC as model crystalline and amorphous substrates, respectively.

As introduced above (Fig. 5), all four cellulolytic *Cellulomonas* LPMOs exhibited a much higher activity on PASC than Avicel under standard conditions containing 0.1% w/v substrate in suspension (Additional file 3: Figure S9a). In detailed time course experiments, *Cfi*LPMO10, *Cfla*LPMO10A, *Cfla*LPMO10B, and *Cfla*LPMO10C all rapidly lost activity over the course of the 16 h assay with both Avicel and PASC (Additional file 3: Figure S9a; *cf.* Additional file 3: Figure S5 for *Cfla*LPMO10A). Supplementation with additional substrate and ascorbic acid could not rescue activity and demonstrated that activity loss was caused by enzyme inactivation rather than substrate or reducing agent availability (Additional file 3: Figure S10). Such inactivation is known to be caused by oxidative damage via futile generation of reactive H_2_O_2_ in the absence of suitable surface area for LPMO binding and catalytic turnover [104]. In keeping with this, inclusion of a tenfold higher concentration of substrate, i.e., 1% (w/v) PASC, greatly prolonged the activity of *Cfla*LPMO10A (Additional file 3: Figure S9b). *Cfla*LPMO10C demonstrated the highest initial activity, generating ca. a twofold higher concentration of oxidized products than any of the other LPMOs at 10 min, yet this also coincided with inactivation by 30 min. Notably, only *Cfi*LPMO10 appeared to be immune to inactivation and continued to generate oxidized products over the entire course of the 16 h incubation. These phenomena were consistently observed at different total enzyme loadings 0.25 μM—1 μM (Additional file 3: Figure S11).

**Fig. 6:**
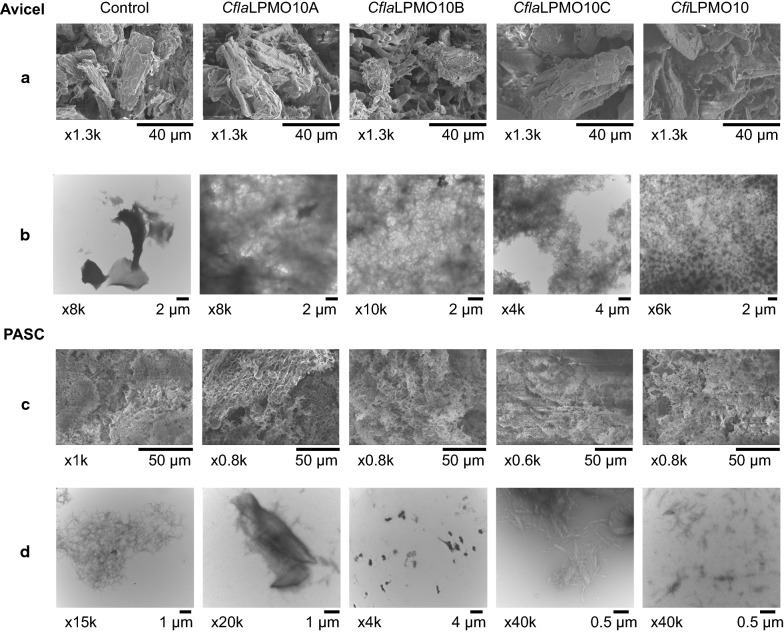
Surface morphology of *Cellulomonas* LPMO-oxidized cellulose visualized under scanning and transmission electron microscopy. **a** SEM of untreated (control) and LPMO-oxidized Avicel. **b** TEM of untreated (control) and LPMO-oxidized Avicel. **c** SEM of untreated (control) and LPMO-oxidized PASC. **d** TEM of untreated (control) and LPMO-oxidized PASC. Scale bars and magnification values are indicated below each representative micrograph

Analysis of soluble products presents only limited insight into the oxidative activity of LPMOs on insoluble cellulosic surfaces. Thus, electron microscopy was used to examine changes in cellulose morphology following LPMO oxidation on Avicel and PASC under identical conditions to those used for quantitative HPLC assays. SEM of untreated Avicel reveals cellulose particles with an estimated size between 40 and 50 µm and a rough surface morphology (Fig. 6a). Under TEM, Avicel appears to be tightly compacted with little particulate dispersion in the aqueous medium (Fig. 6b). Despite comparatively slow release of soluble products in the HPLC-based assays, EM analysis clearly evidenced changes in Avicel morphology with all four LPMOs. A general softening of surface features was observed by SEM, especially for *Cfi*LPMO10 and *Cfla*LPMO10B, concordant with oxidative perturbation of the cellulose surface yielding partially disintegrated, amorphous cellulose. Correspondingly, TEM analysis of LPMO-treated Avicel also demonstrated disintegration of the Avicel particles, as indicated by larger areas containing dispersed, nanocellulosic structures in the aqueous medium. This phenomenon was particularly evident in samples treated with *Cfi*LPMO10 or *Cfla*LPMO10B, concordant with the SEM analysis.

In contrast, the surface of PASC was expectedly composed of very fine cellulose nanofibers and nanoaggregates, which form a thin film structure upon drying. Thus, differences in the surface morphology before and after LPMO oxidation were difficult to distinguish by SEM (Fig. 6c). However, TEM images of LPMO-treated PASC (Fig. 6d) clearly depict the formation of scattered, dense structures indicative of nanofibers in comparison to untreated PASC. These data suggest that the four *Cellulomonas* LPMOs may preferentially attack less-ordered regions of cellulose before addressing the more recalcitrant crystalline nanostructures.

### LPMO combinatorial synergy

**Fig. 7:**
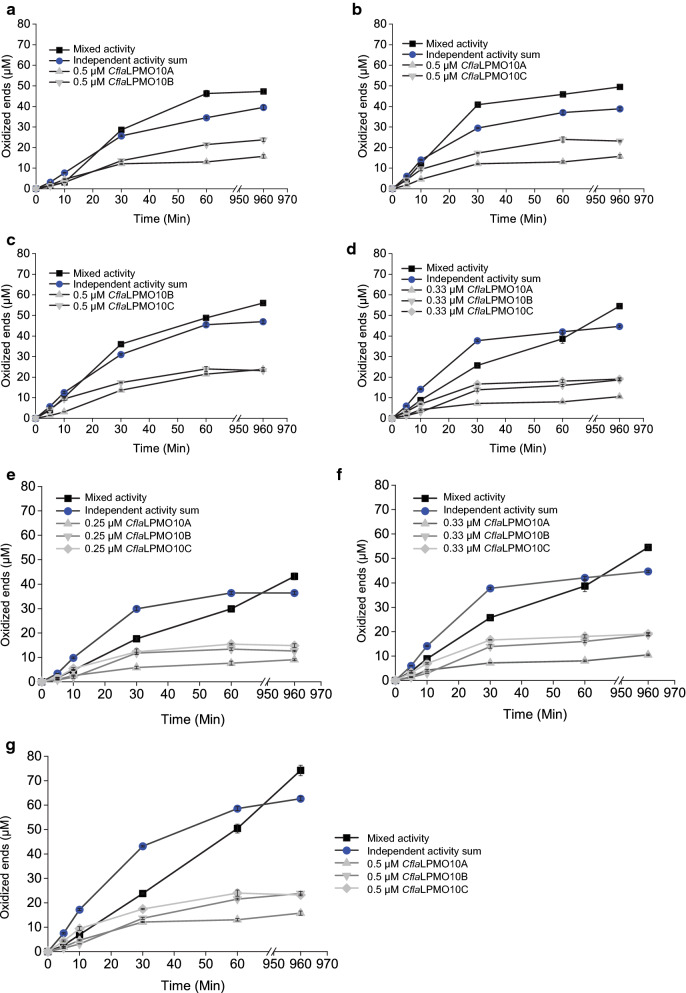
Progress curves of released soluble oxidized products by *C. flavigena* LPMOs incubated in combination and independently. **a**–**d** Double and triple combinations of *C. flavigena* enzymes incubated together at a total enzyme loading of 1 μM compared to the sum total of the independent enzyme proportions at 1 μM. **e**–**g** Boosting effect of a combined mixture of all three *C. flavigena* LPMOs at three different enzyme loading concentrations vs the sum total of independent enzyme proportions at each respective total enzyme load. Total oxidative activity was quantified from simplification of the product profile to cellobionic acid using *T. reesei* cellulase cocktail as described in the results and each assay was performed independently and in triplicate to calculate standard error of the mean

To better understand why *C. flavigena* maintains multiple LPMO-encoding genes within its genome, while *C. fimi* only encodes one, these enzymes were assayed in combination to assess their capacity to collectively boost (or hinder) cellulose degradation. Specifically, LPMOs were assayed on PASC in combinations of two, three, and four using HPLC time course measurements over 16 h. The resulting progress curves show that *C. flavigena* enzymes, in any combination of equal proportions, can effectively boost the release of oxidized products on PASC (Fig. 7). In addition, *C. flavigena* combinations were more active than any individual enzyme at equivalent enzyme loadings. For example*,* 0.33 uM of each of the three *C. flavigena* LPMOs released more oxidized products than 1 µM of any individual *C. flavigena* enzyme (Additional file 3: Figure S12d). Furthermore, the observed boosting effect was noticed to be a result of delayed enzyme inactivation (*cf.* Figure 7 and Additional file 3: Figure S12), as demonstrated by the continuation of oxidized product release up to (and likely beyond) 16 h. As an example, when all three *C. flavigena* LPMOs are combined at equal concentrations (total enzyme loading of 1 μM or 0.33 μM of each enzyme), the quantified concentration of released oxidized product after 16 h was 55 μM. The independent additive release of 0.33 μM of each enzyme on PASC was found to be 45 μM; an increase of just over 20% in oxidative activity (Additional file 3: Fig. 12Sd). This trend is observed at both higher and lower enzyme loadings, suggesting that this synergistic effect is independent of enzyme concentration (Fig. 7). The same trends are not observed when *Cfi*LPMO10 is combined with any of the *C. flavigena* LPMOs. Rather, the addition of any *C. flavigena* LPMO with *Cfi*LPMO10 did not result in prolonged activity, and an overall decrease of soluble oxidized products was observed after 16 h (Additional file 3: Figure S13).

## Discussion

Bacteria from the genus *Cellulomonas*, and *Cellulomonas fimi* in particular, are historically significant organisms in cellulase research [9–14, 39–65, 68]. Here, we have used detailed biochemical analysis to define *Cfla*LPMO10A as a cellulose-active LPMO with strict C-1 specificity (EC 1.14.99.54*)*. In contrast, *Cfi*LPMO10, *Cfla*LPMO10B, and *Cfla*LPMO10C cleaved cellulose at C1 of glucosyl residues in the cellulose chain but also had some capacity to affect cleavage by oxidation at C4 (EC 1.14.99.56). To our knowledge, there has been no AA10 LPMO characterized with exclusive C4 regiospecificity [23], so this may represent a side activity of these three *Cellulomonas* LPMOs. Indeed, the low concentration of C4-oxidized products detected indicates that this activity may be a consequence of incidental cleavage at this position.

Our initial primary structural analysis and homology modeling correctly predicted the substrate specificity and regiospecificity of these enzymes. Of note, we observed that the first aromatic residue within the substrate recognition motifs on the L2 loop (either Tyr or Trp in *Cellulomonas* LPMOs) corresponds to a pattern indicative of regioselectivity. At this position, strict C1-active AA10s have a Tyr residue, while mixed C1/C4-active AA10s have a tryptophan. To our knowledge, this pattern has not been previously reported. Possible exceptions are *Tt*LPMO10A, which contains a Tyr but was reported to have mixed C1/C4 activity [73], and *Kp*LPMO10A, which has a Trp and was reported to have both mixed regioselectivity and activity on both cellulose and chitin [107]. In addition, a similar trend was observed concerning the axial aromatic residue at the active site (Phe or Tyr) [103, 108]. We observed that whereas C1-active AA10 LPMOs contain an axial Phe (regardless of cellulose or chitin specificity), mixed C1/C4-active AA10 LPMOs possess a Tyr at this position. Again, the only exceptions to this pattern among characterized AA10 members are *Tt*LPMO10A and *Kp*LPMO10A [73, 107].

There has recently been significant interest in the identification of thermostable LPMOs, with a particular focus on fungal AA9 members [109, 110]. Interestingly, the cellulolytic LPMOs from the mesophiles *C. fimi* and *C. flavigena* studied here proved to be only moderately thermostable, with pH-dependent *T*_m_ values in the range of 40–60 °C (Additional file 3: Figure S6), yet difficult to inactivate by boiling. Further analysis revealed a strong propensity of these proteins to refold spontaneously following thermal denaturation, which was mediated by the presence of copper ions. Bound copper has been previously associated with modulation of LPMO thermostability [111–114]; given the high affinity of the histidine brace for its ligand, this may nucleate protein refolding. Although the *Cellulomonas* LPMOs are unlikely to find application in sustained high-temperature bioprocesses, their ability to renature upon cooling does not preclude their application to biomass at elevated temperatures followed by cooling.

The analysis of *Cellulomonas* LPMO activity on a panel of cellulosic substrates generally suggested a preference for substrates with high surface area and lower crystallinity. Furthermore, it appears that the stability of these LPMOs is inversely correlated with the rate of catalytic turnover and, by extension, of the potential for futile cycling leading to hydrogen peroxide generation. For example, *Cfla*LPMO10C had the highest initial activity on both PASC and Avicel of the four LPMOs; however, it also succumbed most rapidly to enzyme inactivation.

Many cellulolytic microbes are known to encode multiple LPMOs in their genomes [92, 115, 116], although the biological reasons for this are not well understood. Previous studies on this topic have focused on understanding LPMO regulation by polysaccharide substrates [5, 117], and certainly LPMOs with distinct activity profiles toward cellulose, hemicellulose, and other polysaccharides have been identified [23–29]. The need to produce multiple LPMOs of apparently similar activity is less obvious, but as for hydrolytic cellulases, there is some evidence for functional synergy among these enzymes [92]. Likewise, our combinatorial LPMO experiments demonstrated synergy between the three *C. flavigena* LPMOs. Strikingly, synergy between these LPMOs was not observed in any combination with the lone *C. fimi* LPMO10; thus, all cellulolytic LPMOs—even from closely related species—are not created equal. The production of multiple LPMOs in response to specific substrates is not a simple gene dosage phenomenon, but rather serves to deliver a suite of complementary enzymes acting together to enable more efficient saccharification of recalcitrant paracrystalline cellulose.

We did not pursue the characterization of the remaining AA10 member from *C. flavigena* because our bioinformatic analysis strongly indicated that this protein is likely to be a chitin-active LPMO (EC 1.14.99.53) and was thus outside the scope of this study. Nonetheless, we note that facultative chitin utilization by a *Cellulomonas* bacterium was previously reported [118], and that the genomes of *C. flavigena* and *C. fimi* encode putative chitin-targeting GH23 and CBM50 members. By analogy with the comprehensive study of cellulolytic GH and LPMO in *Cellulomonas* species, exploration of the full hydrolytic and oxidative chitin-degrading system of these bacteria is worthy of future pursuit.

## Conclusions

The comprehensive elucidation of the specificity and mechanism of the complete repertoire of carbohydrate-active enzymes encoded by an organism (the “CAZome”) is essential to fully understand the roles that an environmental microorganism fulfills in the global carbon cycle. The present study advances understanding of the cellulose utilization machinery of historically important *Cellulomonas* species beyond hydrolytic enzymes to include lytic cleavage of the world’s most abundant biopolymer. Additionally, this work contributes to the broader mapping of enzyme activity in Auxiliary Activity Family 10 and provides new biocatalysts for potential applications in biomass modification.

## Materials and methods

### Bioinformatic analyses

Full-length protein sequences of biochemically characterized LPMO enzymes were obtained from GenBank via hyperlinks in the CAZy database [68] (http://www.cazy.org/AA10_characterized.html) and were aligned using BLASTP to identify *C. fimi* and *C. flavigena* AA10 catalytic module boundaries. Following truncation to remove predicted carbohydrate-binding modules (CBM) or other domains at the C termini, the *Cellulomonas* AA10 sequences were aligned with those of the characterized AA10 members and three characterized AA9 sequences (comprising an outgroup) using MUSCLE [119] within MEGA [120] and manually realigned to preserve structurally homologous residues (Additional file 1). A maximum-likelihood phylogeny (Additional file 2) was subsequently generated using RAXML 8.2.10 within the CIPRES Science Gateway v3.1 [121] with the following settings: JTT matrix-based nucleotide substitution model [122] of 25 discrete rate categories and rapid bootstrapping with automatic halting enabled (702 bootstraps). Figtree was used to visualize the resulting phylogenetic tree (http://tree.bio.ed.ac.uk/software/figtree). Three-dimensional homology models were generated using Phyre^2^ [81].

### Gene cloning and protein production

cDNA encoding the native signal peptide and catalytic domain of *Cfla*LPMO10A (encoded by genome locus Cfla_0175, GenBank accession ADG73094.1), *Cfla*LPMO10B (encoded by genome locus Cfla_0172, GenBank accession ADG73091.1), *Cfla*LPMO10C (encoded by genome locus Cfla_0316, GenBank accession ADG73234.1), and *Cfi*LPMO10 (encoded by genome locus Celf_0270, GenBank accession AEE44415.1) were amplified from gDNA of *Cellulomonas flavigena* DSM 20,109 and *Cellulomonas fimi* ATCC 484, respectively. PCR products were purified from agarose gel using a Qiaquick Gel Extraction Kit (Qiagen, Hilden, Germany, P/N: 28,115). The cDNA were subsequently inserted into pET29b(+) vectors using NdeI/XhoI sites for *Cfi*LPMO10 (Celf_0270) and *Cfla*LPMO10B (Cfla_0172) and NdeI/NotI sites for *Cfla*LPMO10A (Cfla_0175) and *Cfla*LPMO10C (Cfla_0316) using restriction enzymes purchased from NEB (Ipswitch, MA, P/N: R0111, R0146, and R0189 for NdeI, XhoI and NotI, respectively). Correctly cloned constructs were verified by Sanger sequencing (GENEWIZ).

All four pET constructs were transformed into Rosetta™-(DE3) pLysS *E. coli* cells (Millipore Sigma, Oakville, ON Canada, P/N: 70,956). Protein expression was induced in LBE-5052 autoinduction medium [123] containing 50 μg/ml kanamycin (Millipore Sigma, P/N: 10106801001) and 35 μg/ml chloramphenicol (Millipore Sigma, P/N: C1919) and grown in shake flasks for 26 h at 25 °C at 250 rpm. Media were then centrifuged at 18,600 × *g* for 20 min at 4 °C in a Beckman Coulter Avanti J-E Highspeed Centrifuge (Brea, CA, USA). The supernatant was collected and treated with Roche 1X cOmplete, Mini EDTA-free Protease Inhibitor cocktail (Millipore Sigma, P/N: COEDTAF-RO) and concentrated to 20 ml using a 10,000 kDa cut-off Vivaflow 200 PES membrane from Sartorius (Göttingen, Germany, P/N: VF20PO). The pH of the concentrated supernatant was adjusted to 8 using NaOH, filtered through a 0.22 μm PES membrane and injected on a 1 ml Ni–NTA prepacked column from GE Healthcare (Chicago, IL, USA, P/N: 17–5247-01) pre-equilibrated in binding buffer (20 mM Tris–HCl pH 8, 500 mM NaCl and 10 mM imidazole). The column was subsequently washed with 10 mL of binding buffer and mature His-tagged LPMO proteins were eluted using a linear gradient of 0 to 100% elution buffer (20 mM Tris–HCl pH 8, 500 mM NaCl, and 500 mM imidazole) over 12 min. The elution fractions were collected and incubated with 1 mM CuCl_2_ (Millipore Sigma, P/N: 222,011-250G) for 1 h at room temperature and subsequently desalted via size-exclusion chromatography using a 16 × 600 mm column of Sephadex G-25 (GE Healthcare, P/N: 17003301) pre-equilibrated in storage buffer (20 mM Tris–HCl buffer pH 8), flash frozen in aliquots, and stored at -70 °C until use. Protein fidelity was confirmed using intact protein MS, essentially as previously described [124]; correct cleavage of the signal peptide to yield the N-terminal His-1 required for catalytic activity was observed in all cases; however, limited proteolysis of the C-terminus was observed for *Cfla*LPMO10A and *Cfi*LPMO10 (Additional file 1: Figure S1A and S1D).

### Carbohydrates

Avicel, chitin, and chitosan substrates were purchased from Sigma Aldrich (St. Louis, MO, USA). Starch and glucose were purchased from Fisher Scientific (Hampton, NH, USA). Cellobiose was purchased from Acros Organic (Morris Plains, NJ, USA). Tamarind xyloglucan, barley mixed-linkage β-glucan, carboxymethyl cellulose, hydroxyethyl cellulose, beechwood xylan, curdlan, cellotriose, cellotetraose, cellopentaose, and cellohexaose were purchased from Megazyme International (Bray, Ireland). Northern bleached softwood Kraft pulp (NBSKP) was donated by Canfor Pulp (Vancouver, BC, Canada). Bacterial cellulose was grown and harvested from *Komagataeibacter xylinus* (i.e., *Acetobacter xylinum*, *Gluconacetobacter xylinus*) following a previously published protocol [125]. Cellulose nanocrystals (CNC) were produced from acid hydrolysis of NBSKP and cellulose nanofibrils (CNF) were produced from NBSKP by low-consistency refining at the UBC Pulp and Paper Centre. Phosphoric acid-swollen cellulose (PASC) was prepared from Avicel following a previously published protocol [126].

C1-oxidized cello-oligosaccharide standards were prepared using *Podospora anserina* cellobiose dehydrogenase (CDH), kindly provided by Dr. Jean Guy Berrin (INRA, Marseilles, France) [127]. 1 μM of CDH was added to a 2 mM of each cello-oligosaccharide (Glc_2_–Glc_5_) in 50 mM sodium acetate buffer, pH 5, followed by incubation at 50 °C. Full conversion was achieved by supplementation of additional enzyme every 24 h for 3 days.

### Protein denaturation analysis

Thermofluor denaturation assays were performed in 7500 Fast Real-Time PCR system (Applied Biosystems) based on a previously published protocol [128]. MicroAMP Fast Reaction 8-strip tubes (Thermo Fisher, Waltham, MA, USA, P/N: 4358293) and strip caps (Thermo Fisher, P/N: 4323032) were used as reaction vessels in which 5 μM enzyme was incubated with 20 mM of appropriate buffer solution and SYPRO Orange diluted to a final 10X concentration from a 5000X stock as supplied by the manufacturer (Invitrogen, Carlsbad, CA, USA, P/N: S6650). 1 mM D-penicillamine (Alfa Aeser, Heysham, Lankashire, UK, P/N: A11446) was used for copper chelation experiments. Thermal denaturation curves were produced by first holding the reaction at 10 °C for 6 min followed by a constant ramp rate of 0.85 °C per minute (1%) until 95 °C and held for 1 min followed by a final drop to 10 °C and held for 10 min. 7500 Software v.2.3 (Applied Biosystems) was used to collect fluorescence data.

Differential scanning calorimetry (DSC) experiments were performed on a Microcal™ VP-Capillary DSC (Malvern Panalytical, Westborough, MA, USA) based on a previously published protocol [129]. 20 μM enzyme in 200 μL containing appropriate buffers (50 mM sodium acetate pH 3.5, 50 mM BisTris pH 5, 50 mM BisTris pH 7, and 50 mM glycine pH 10) were degassed under vacuum for 10 min before loading into sample cell (cell volume 133 μL). Background scans were performed prior to each experiment using buffer blanks to generate normalized data. The calorimeter was programmed to ramp to 100 °C at a scan rate of 60 °C per hour before cooling to 10 °C and holding for 30 min before starting the second scan. Data analysis and curve fitting were performed using Origin Pro software.

### LPMO activity assay

The oxidation of insoluble cellulosic substrates by *Cellulomonas* LPMOs was performed at 37 °C with shaking at 1000 rpm in an Eppendorf Thermomixer C (Hamburg, Germany) for up to 16 h. Typical assays were performed in 300 μL with 0.1% w/v substrate using 50 mM citrate–BSA buffer, containing 0.02% bovine serum albumin (VWR, Radnor, PA, USA, P/N: 97061-420). 1 mM ascorbic acid (Fisher Bioreagents, P/N: B351) was added last to start the reaction and enzymatic reactions were stopped by the removal of substrate via centrifugal filtration through 0.2 μm cellulose acetate spin filters (VWR, P/N: 2994-752). For peroxidase activity assays, 100 μM of hydrogen peroxide was added at the start of the experiment. Soluble oxidized oligosaccharides were subsequently detected via High-Performance Anion-Exchange Chromatography coupled with Pulsed Amperometric Detection (HPAEC-PAD) as described below. For quantification of LPMO activity, *T. reesei* cellulase cocktail (Celluclast, Sigma-Aldrich, P/N: C2730-30) was used to hydrolyze released soluble cello-oligosaccharides into glucose, cellobiose, and cellobionic acid (note: gluconic acid was not observed in these hydrolyzed samples). Total oxidation activity was calculated from quantification of oxidized cellobionic acid through standard calibration curves. In general, 1 unit of Celluclast was added to 100 μL of LPMO assay supernatant and incubated at room temperature for 1 h before analysis by HPAEC-PAD.

### Product analysis by HPAEC-PAD

Soluble cello-oligosaccharides and corresponding oxidation products were analyzed by HPAEC using an ICS-5000 system (Dionex, Sunnyvale, CA, USA) coupled to a gold electrochemical detector (Dionex, P/N: 072044) for PAD. The oligosaccharide separation methodology was designed based on a previously published protocol [130]: 25 μL of sample were injected on a CarboPac PA200 2 × 250 mm analytical column (Dionex, P/N: 062896) with a CarboPac PA200 2 × 50 mm guard (Dionex, P/N: 062895) column maintained at 30 °C. Oligosaccharide separation was achieved at a constant flow rate of 0.5 mL/min. Initial column equilibration for 10 min with an isocratic flow of 0.1 M NaOH, followed by a linear gradient toward 0.25 M NaOAc over 30 min and then a stepwise increase to 0.9 M NaOAc for 1 min as a column cleaning step.

### Product analysis by MALDI-MS

Matrix-assisted laser desorption/ionization time-of-flight (MALDI-TOF) mass spectrometry was performed using a matrix of 9 mg/ml 2,5-dihydroxybenzoic acid (DHB) (Sigma-Aldrich, P/N: 149357) dissolved in 0.2% trifluoroacetic acid (TFA). 2 μL of the DHB matrix was mixed with 2 μL assay supernatant and placed on a Bruker MTP 384 ground steel MALDI plate (Bruker Daltonics, Bremen, Germany P/N: 8280784) and dried under a stream of air prior to data collection. Experiments were performed on a Bruker Daltonics Autoflex system (Billerica, MA, U.S.A.). The data acquired over the range of *m/z* 0 to 3000 were collected in positive-ion mode by averaging 2000 laser shots using the lowest energy sufficient to obtain adequate signal-to-noise ratios.

### Electron microscopy

Transmission electron microscopy (TEM) samples were first prepared by drop casting 2 µL of enzymatic reactions containing 0.025% of cellulose substrate on a 200-mesh carbon-coated copper TEM grid (TED PELLA, Inc., CA, USA). The grids were air dried at room temperature, followed by application of 5 µL drops of aqueous uranyl acetate (2%) to the dry grids. After 1 min, the excess stain was removed by capillary action and gentle blotting, followed by air drying at room temperature. The negatively stained specimens were imaged using a Hitachi H7600 transmission electron microscope (Hitachi, Ltd., Tokyo, Japan) with an accelerating potential of 80 keV. Images were captured on a 5 MPAMT XR50 CCD camera (Advanced Microscopy Techniques Corp., MA, USA). The magnification bars on the images were calibrated using replica grating.

Scanning electron microscopy (SEM) samples were prepared by lyophilization of enzymatic reactions containing 0.1% cellulose substrate for 72 h, followed by sputter coating with 10 nm of platinum or gold before imaging (108 auto/SE, Cressington Scientific Instruments, UK). Imaging was performed using a Hitachi S-4700 Field Emission scanning electron microscope (Hitachi, Ltd., Tokyo, Japan) with an accelerating potential of 5 keV.

## Supplementary Information

**Additional file 1:** Multiple sequence alignment of LPMOs.

**Additional file 2:** Maximum-likelihood phylogenetic tree in Newick format.

**Additional file 3:** Additional figures and tables.

## Data Availability

All data generated or analyzed during this study are included in this published article and its supplementary information files:
