# Supplementary material for: Four cellulose-active lytic polysaccharide monooxygenases from Cellulomonas species
Source: Biotechnol Biofuels. 2021 Jan 23;14:29. doi: 10.1186/s13068-020-01860-3 (PMC7828015; doi:10.1186/s13068-020-01860-3)
Supplement: Supplementary file 3 — Additional file 3: Additional figures and tables. [file 13068_2020_1860_MOESM3_ESM.docx]

## Supplemental Figures


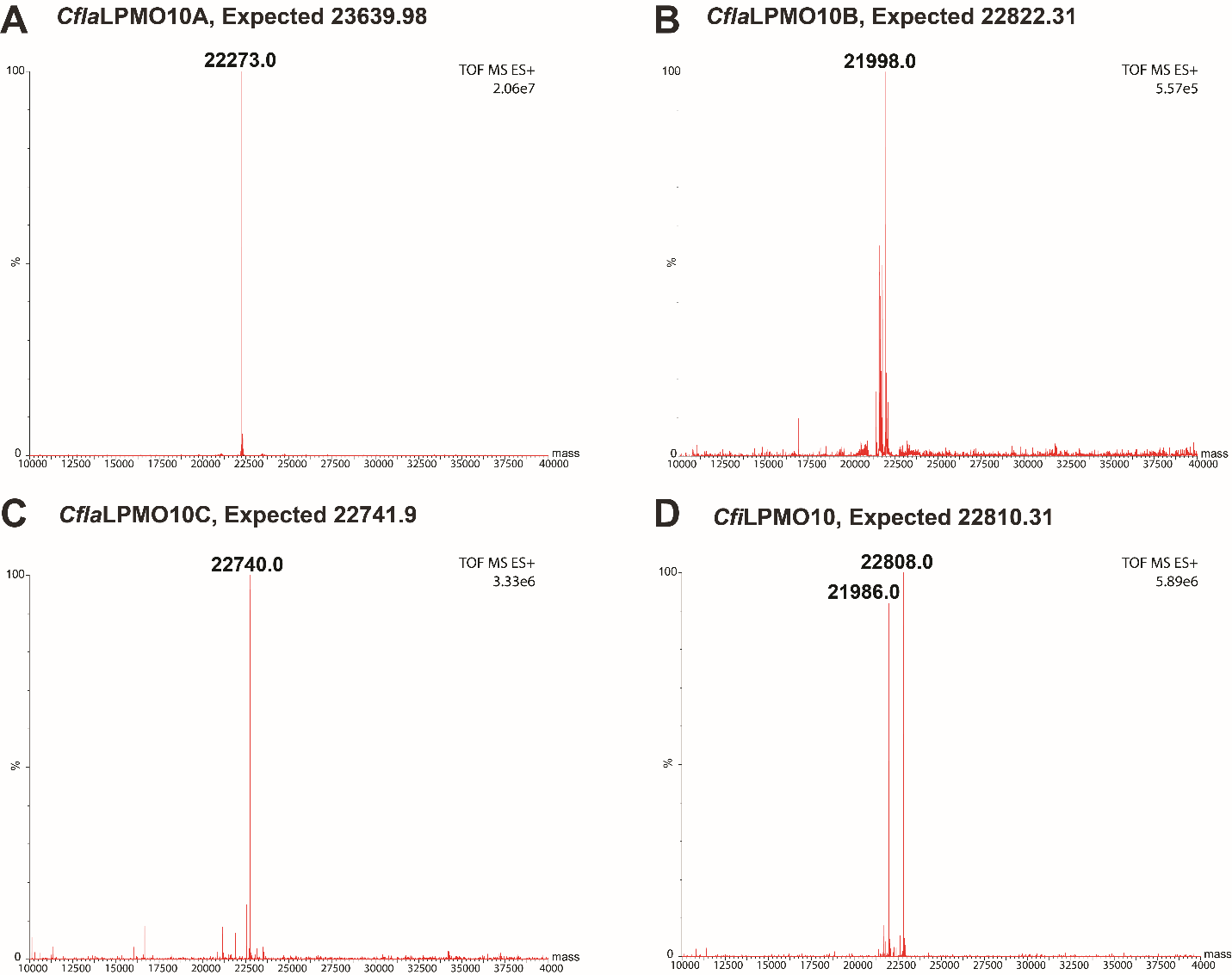


### Supplemental Figure S1. Intact MS spectrum of purified *Cellulomonas* LPMOs. (A) Spectrum of *Cfla*LPMO10A. (B) Spectrum of *Cfla*LPMO10B. (C) Spectrum of *Cfla*LPMO10C. (D) Spectrum of *Cfi*LPMO10. The expected sizes of each LPMO is annotated above each respective panel.

**
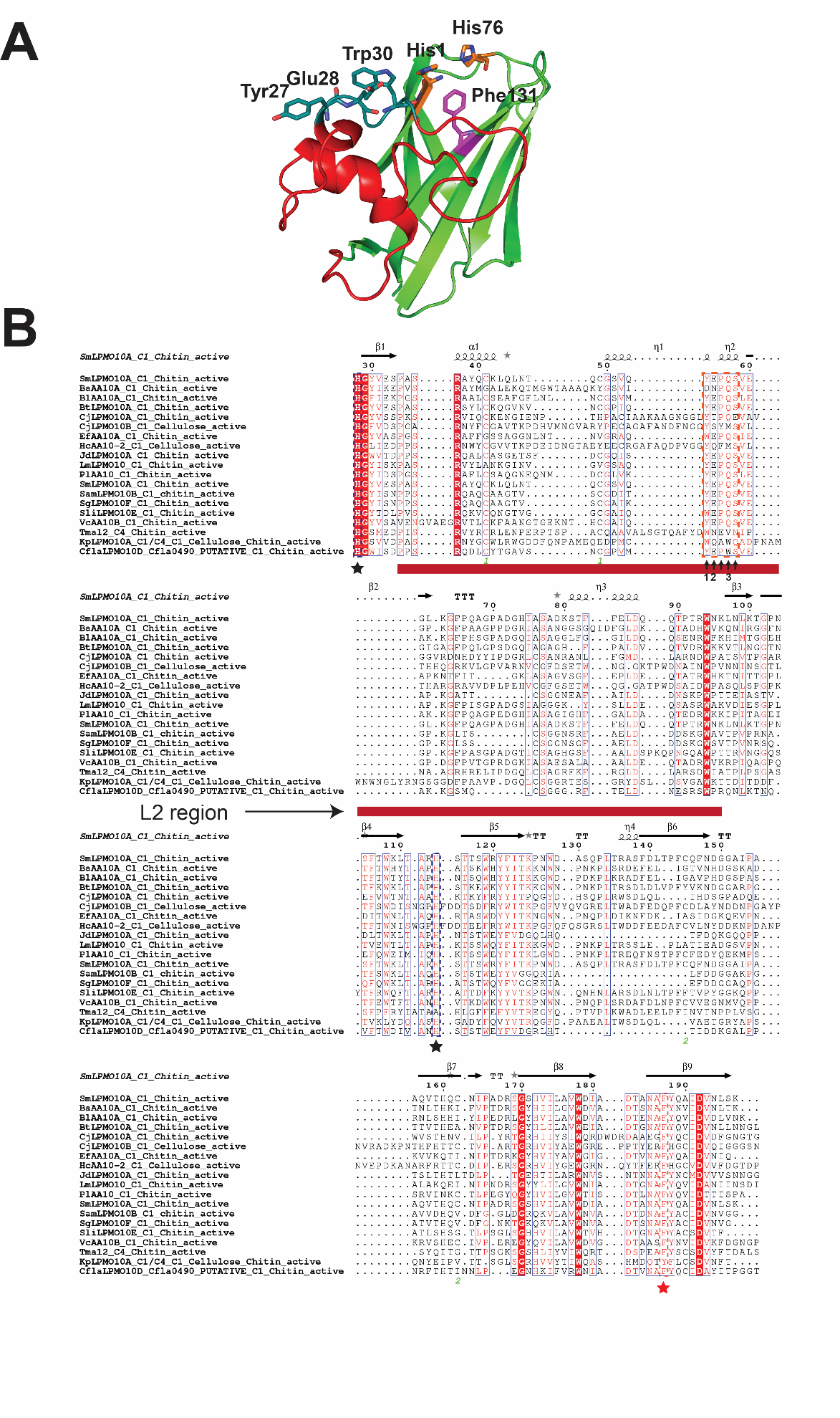
**

### Supplemental Figure S2. Homology model and primary sequence alignment of putative chitin-active CfLPMO10D from *Cellulomonas flavigena* and all known chitin-active AA10 LPMOs. (A) Cartoon representation of three-dimensional homology model of *Cfla*LPMO10D generated using the Phyre^2^ server [73]. The active-site histidine residues are depicted as orange sticks, the three conserved residues of the cellulose-active motif are depicted as yellow sticks, the axial tyrosine or phenylalanine residues are depicted as turquoise or pink sticks respectively, and the catalytic glutamate of *Cfla*LPMO10A is depicted as a teal stick. All residues are numbered starting from the first residue (His) in the mature protein sequence. The L2 region is coloured red to distinguish from the immunoglobulin-like β-sandwich core coloured in green. (B) Sequence and secondary structure alignment of chitin-active AA10 LPMOs compared to LPMO10A from *S. marcescens.* Important active site and substrate targeting residues are indicated on the alignment. The chitin-active motif is denoted by a orange dotted box and the positioning of the conserved residues are numbered 1-3. The two active site histidine residues are denoted by black star symbols. The red dotted box and star denote the position of the axial aromatic residue.

**
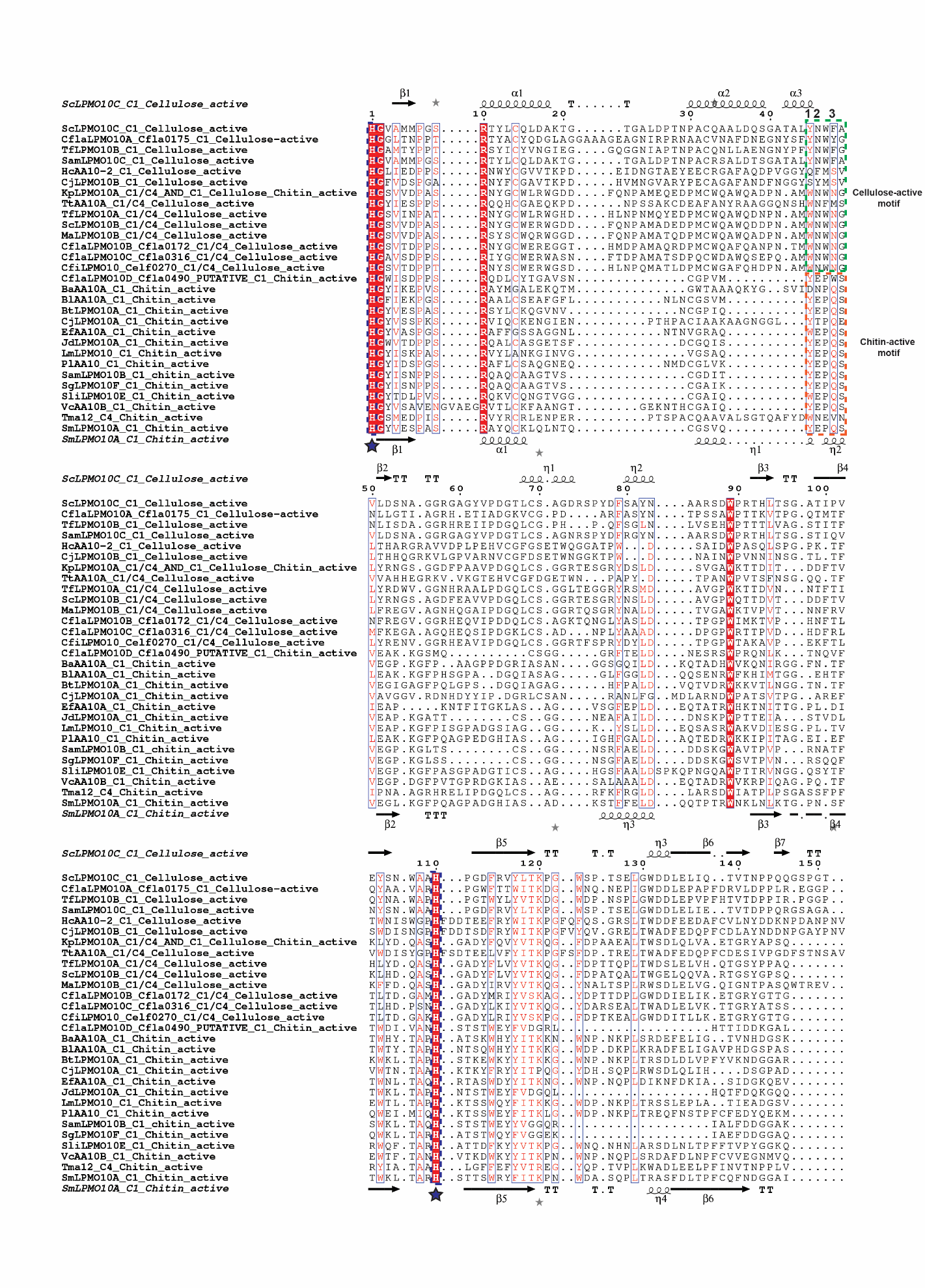
**

**
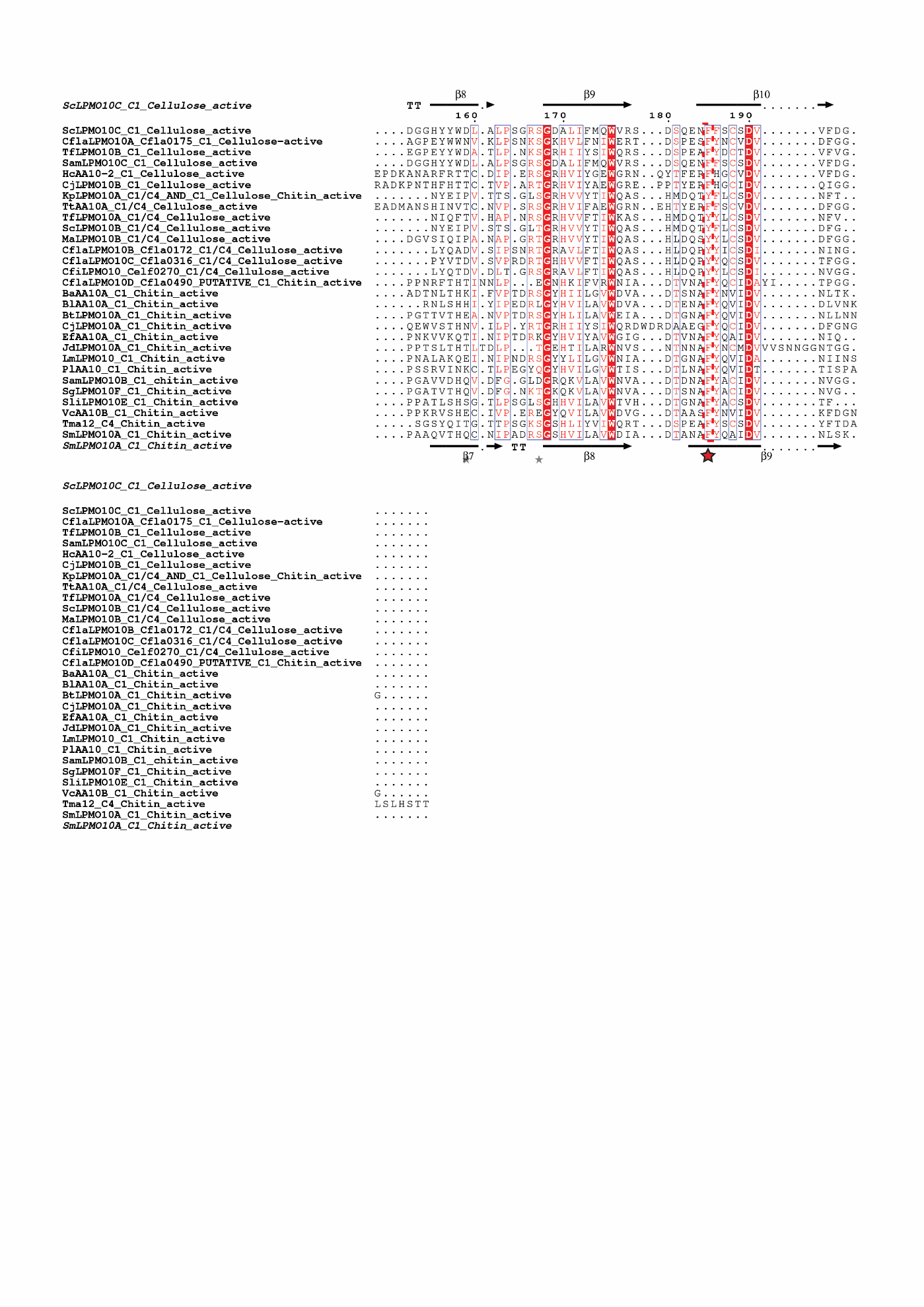
**

### Supplemental Figure S3. Sequence alignment of all currently characterized AA10 LPMOs. The cellulose-binding and chitin binding motifs are highlighted in green and orange dotted boxes respectively and the important conserved residues are numbered above. The blue dotted box and blue star indicates the position of the catalytic histidines involved in copper coordination The red dotted box and red star indicates the axial aromatic residue (Phe or Tyr) of the active site. Muscle was used to align the sequences and the alignment was visualized using Espript 3.0 (<http://espript.ibcp.fr/ESPript/ESPript/>).


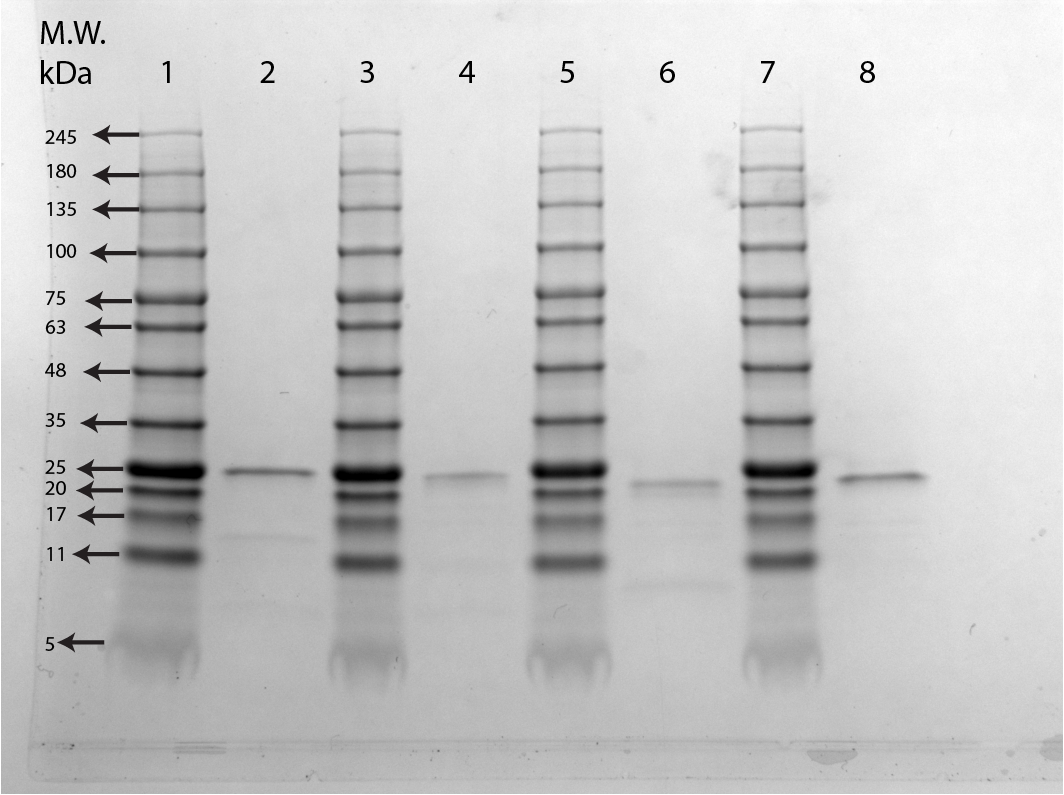


### Supplemental Figure S4. SDS-PAGE analysis of purified *Cellulomonas* LPMOs. Lanes 1, 3, 5, and 7: Protein molecular weight (MW) markers (kDa). Lane 2: 1 μg *Cfla*LPMO10A (expected MW 23.6 kDa). Lane 4: 1 μg *Cfla*LPMO10B (expected MW 22.8 kDa). Lane 6: 1 μg *Cfla*LPMO10C (expected MW 22.7 kDa). Lane 8: *Cfi*LPMO10 (expected MW 22.8 kDa).


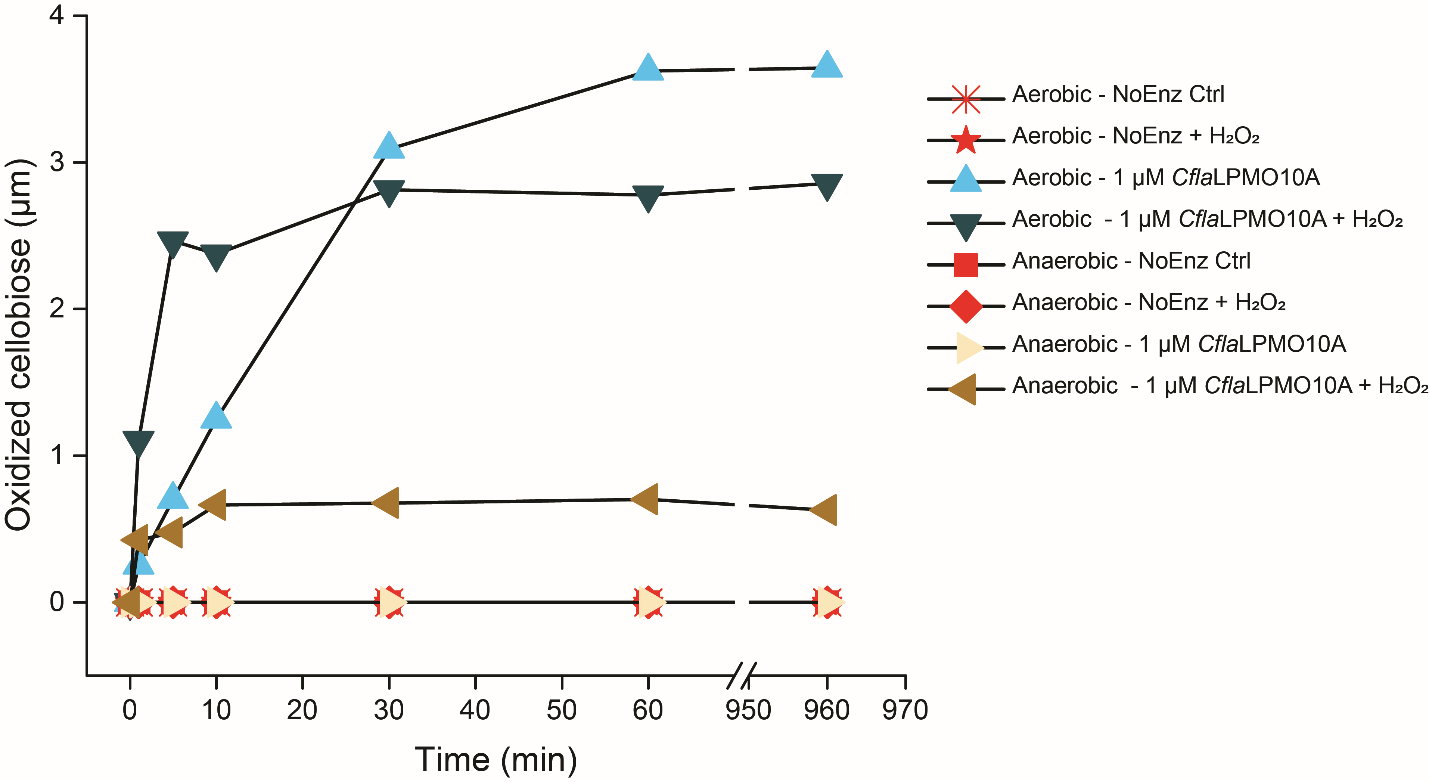


### Supplemental Figure S5. Progress curves of CfLPMO10A on PASC under both aerobic and anaerobic conditions in the presence and absence of hydrogen peroxide. 1 μM *Cfla*LPMO10A was incubated with 0.1% PASC and 1 mM ascorbic acid over 16 hours. In LPMO assays containing hydrogen peroxide, 100 μM of H_2_O_2_ was added at the same time as 1 mM ascorbic acid. Note: Distinct from other quantitative experiments in which *T. reesei* Celluclast cocktail was used, here 100 nM CenD endoglucanase from *Cellulomonas fimi* [14] was used to convert soluble LPMO products to oxidized cellobiose, oxidized cellotriose and oxidized cellotetraose, whereupon oxidized cellobiose was quantified as a proxy for total activity. Each timepoint represents the average of three independent assays measured singly by HPLC, with error bars indicating the standard error of the mean.

###
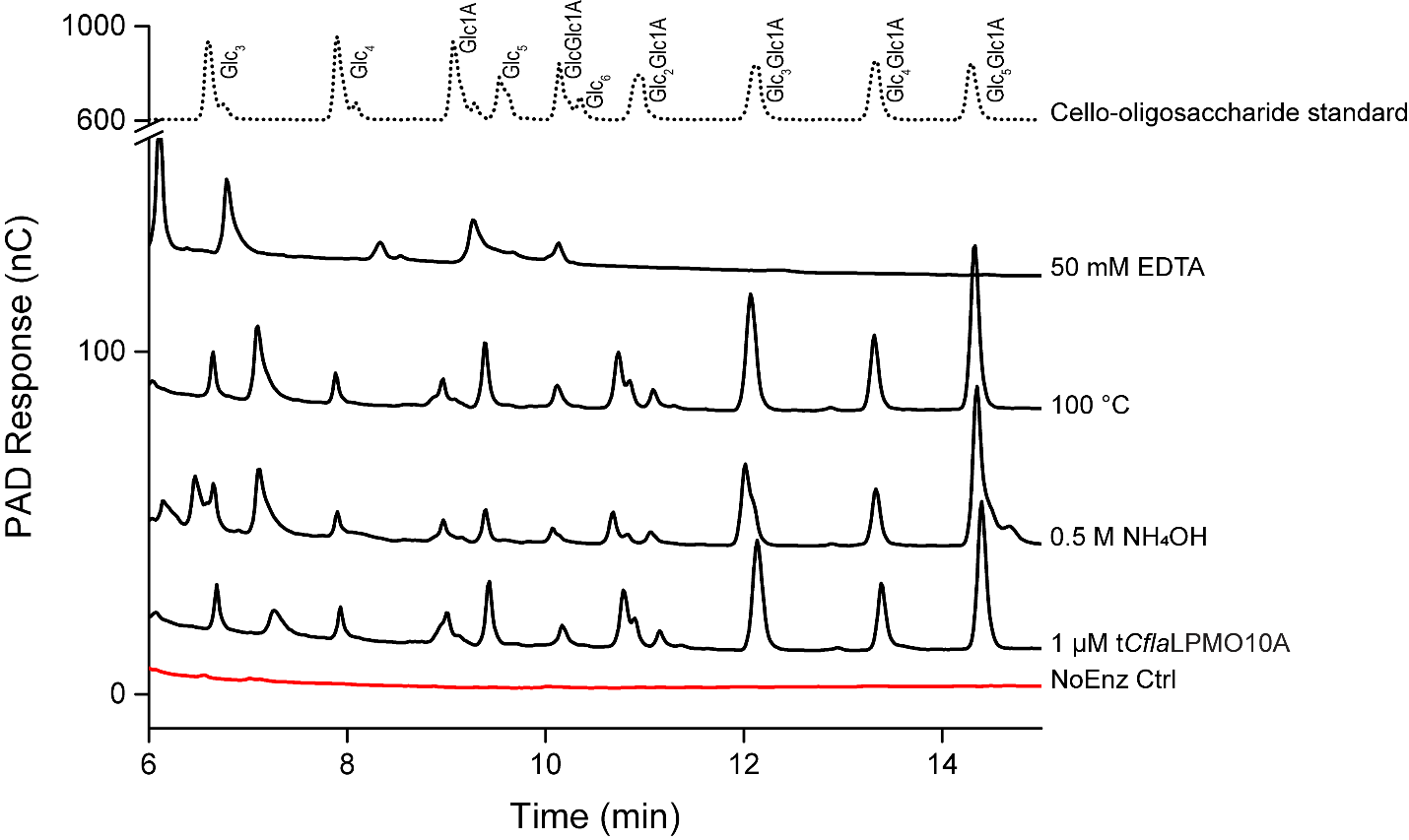
Supplemental Figure S6. Product profile of *Cfla*LPMO10A on PASC following treatment at different deactivating conditions. Each reaction contained 1 μM LPMO, 0.1% PASC and 1 mM ascorbic acid and incubated at 37°C in a shaking incubator. Released soluble products analyzed on HPAEC-PAD. Each deactivating condition is specified beside the corresponding chromatogram. The negative control (NoEnz Ctrl) is shown in red.

**
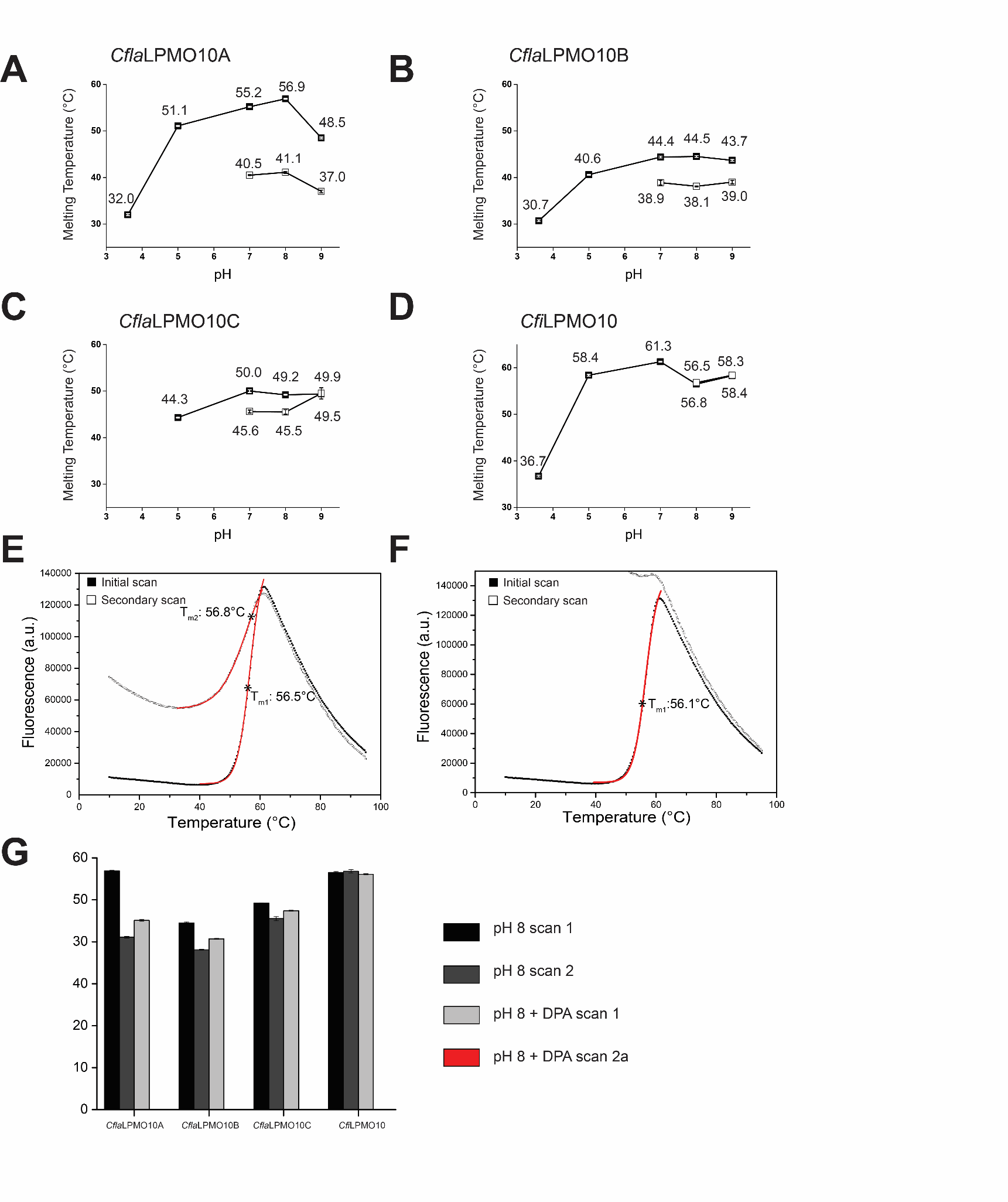
**

### Supplemental Figure S7. Thermostability study of *Cellulomonas* LPMOs. A-D: DSF melting data for each *Cellulomonas* LPMO (indicated above each panel) of 5 μM at different pH conditions. Secondary melting scan data was collected following cooling to 10 °C after initial melting scan. Protein melting temperature (*T*_m_) is indicated beside each pH condition; values represent the standard error of the mean for three independent measurements. E-F: Representative Thermofluor melt curve of *Cfi*LPMO10 at pH 8. Secondary scan data is depicted in blank white boxes. G: Observed *T*_m_ at pH 8 with and without DPA. No refolding was observed when copper was chelated using DPA. 20 X SYPRO Orange dye was used in each Thermofluor assay. All curve fitting and data analysis was performed on Origin Pro.


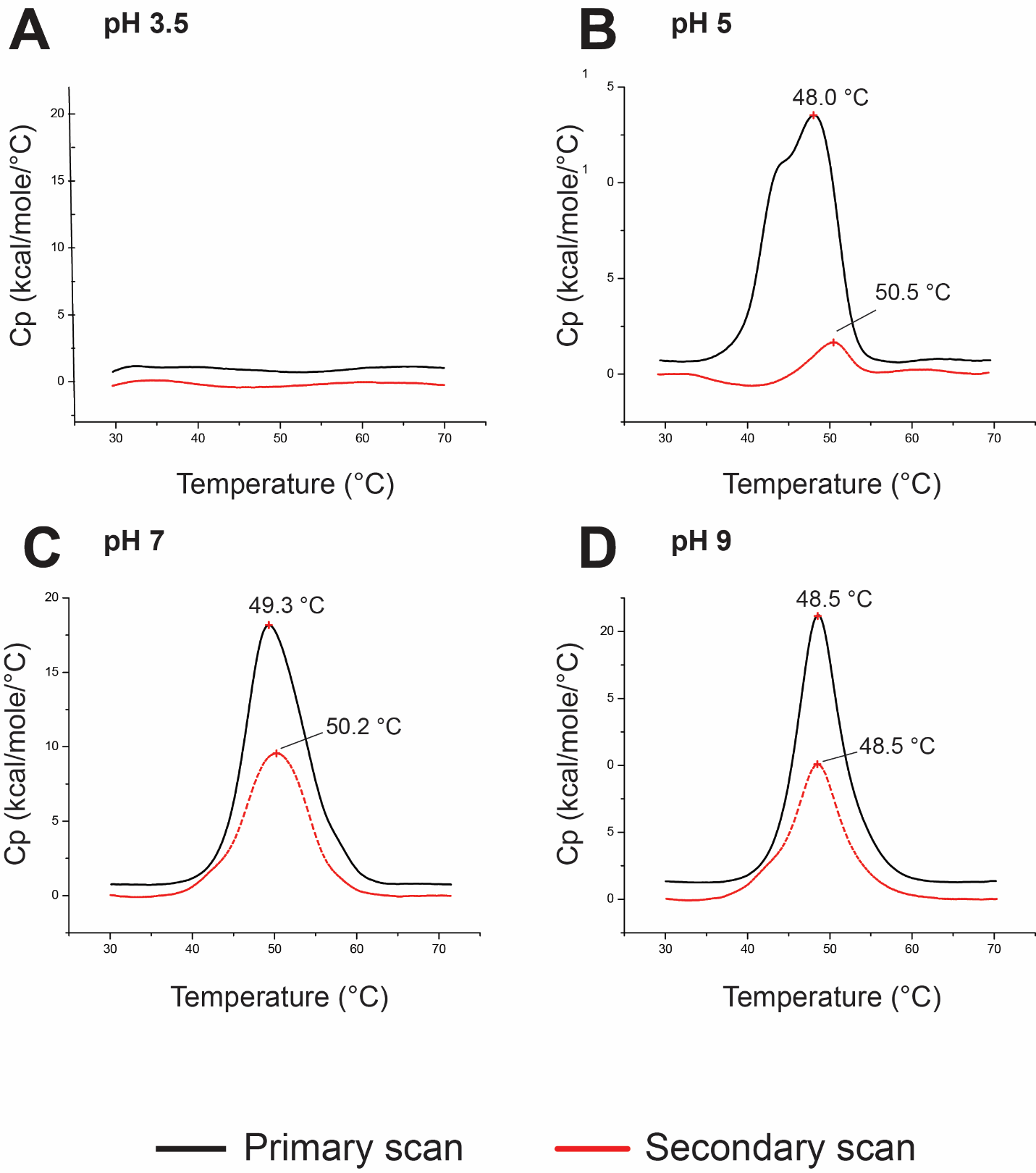


### Supplemental Figure S8. Normalized differential scanning calorimetry curves of *Cfla*LPMO10A denaturation at different pH. (A) Thermogram of *Cfla*LPMO10A in 50 mM sodium acetate pH 3.5 (B) Thermogram of *Cfla*LPMO10A in 50 mM Bis-Tris pH 5.0 (C) Thermogram of *Cfla*LPMO10A in 50 mM Bis-Tris pH 7.0 (D) Thermogram of *Cfla*LPMO10A in 50 mM glycine pH 9. The ramp rate was 100°C/hour. The black line indicates primary melting scan and the red line indicates secondary melting scan. The melting temperature, *T*_m_, is indicated above each curve.


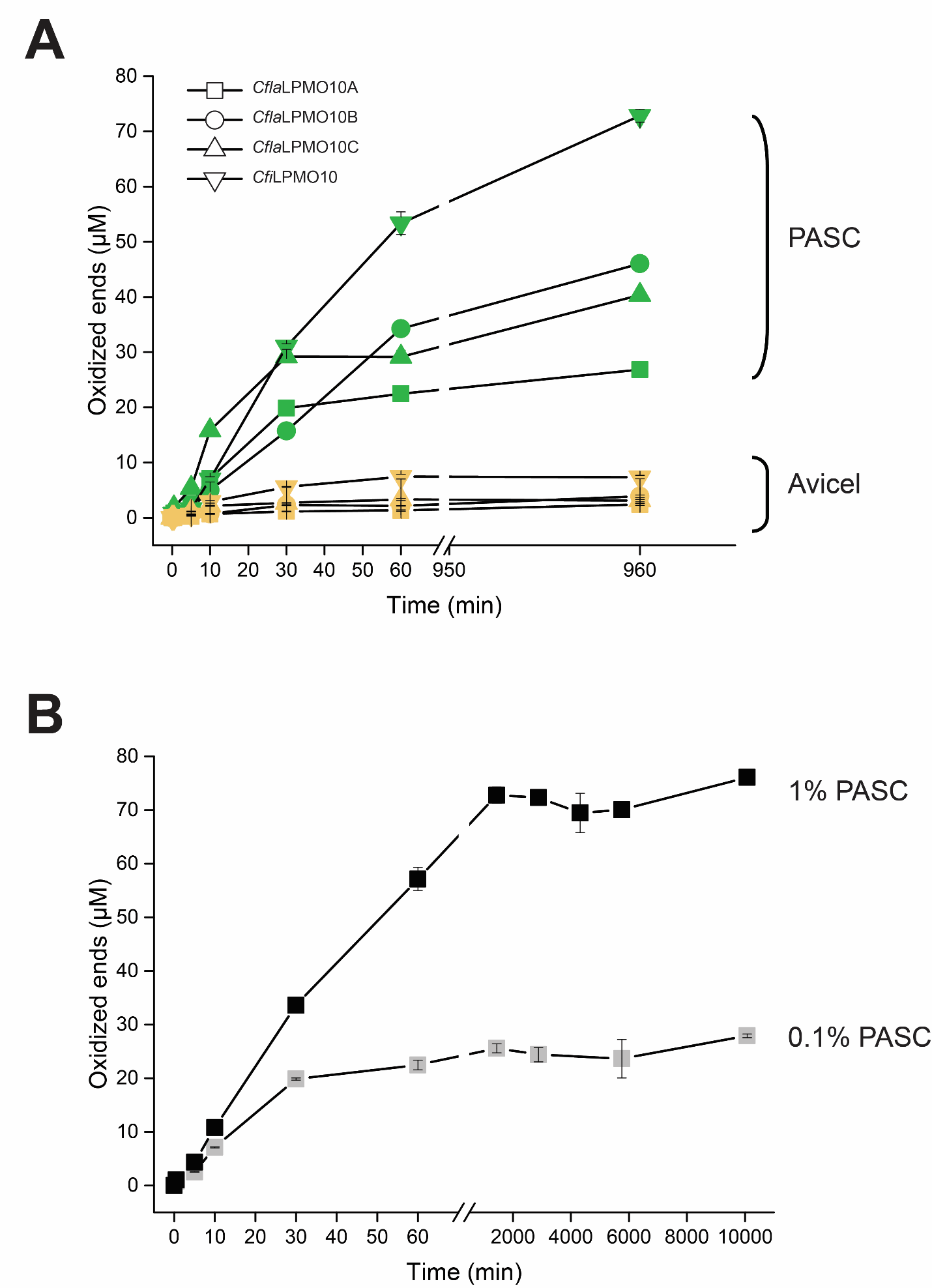


### Supplemental Figure S9. Progress curves of released C1-oxidized ends of *Cellulomonas* LPMOs on cellulose over time. (A) Comparative release of C1-oxidized ends on PASC and Avicel over 16 hours for all four *Cellulomonas* LPMOs at 1 μM enzyme loading and 0.1% substrate. (B) Effect of substrate concentration on *Cfla*LPMO10A catalytic progress over 1 week at 1 μM enzyme concentration. Each reaction contained was performed in 50 mM bis-tris pH 6.8, 1 mM ascorbic acid and incubated at 37°C in a shaking incubator. Total oxidized ends were obtained by quantifying cellobionic acid by HPLC following hydrolysis of soluble products with *T. reesei* Celluclast enzyme cocktail. Each each timepoint represents the average of three independent assays measured singly by HPLC, with error bars indicating the standard error of the mean.


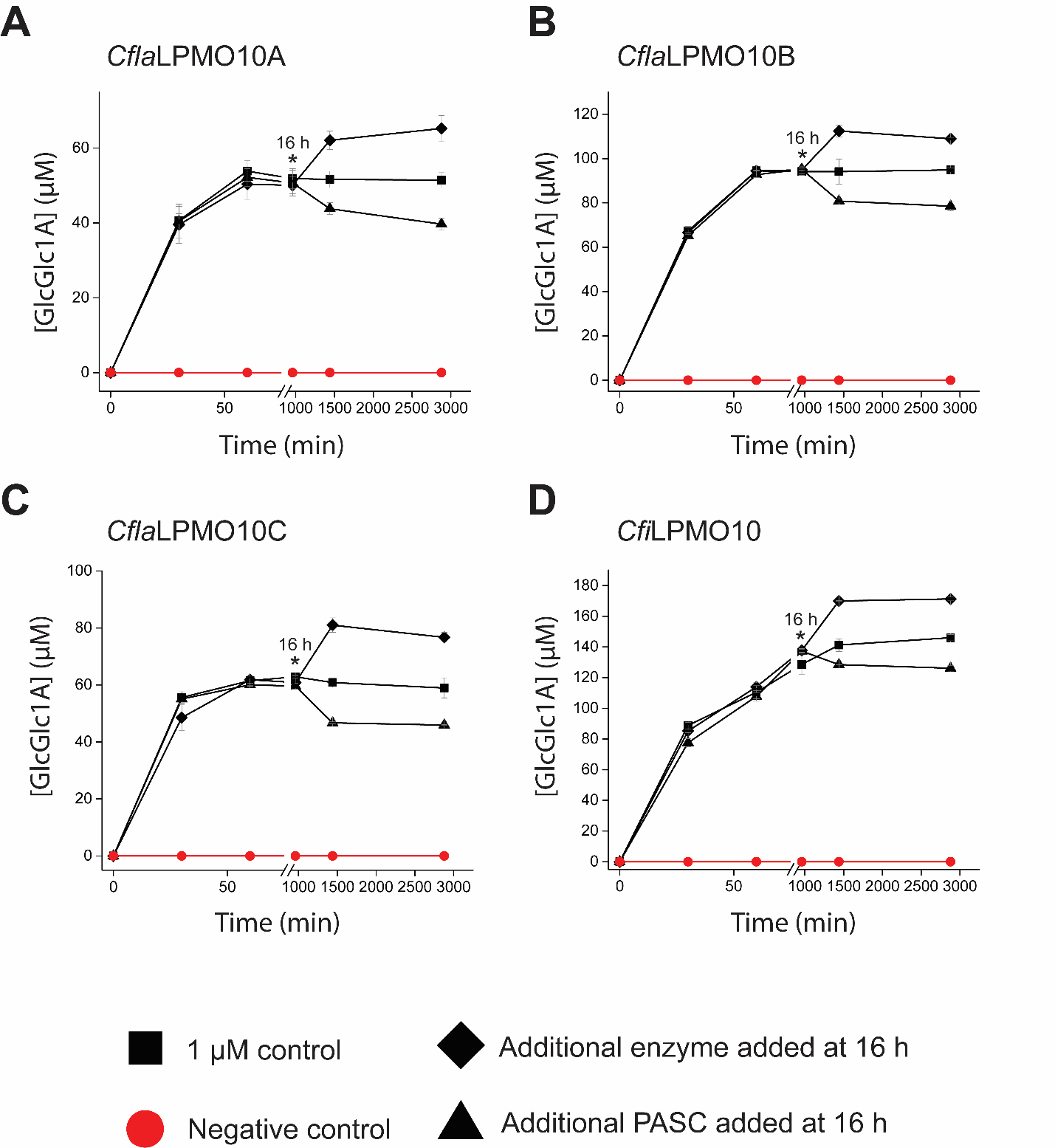


### Supplemental Figure S10. The effect of additional enzyme or PASC substrate introduced at 16 h on *Cellulomonas* LPMO progress curves over time. A-D: 1 μM of each enzyme was incubated with 0.1% PASC and 1 mM ascorbic acid for 16 hours followed by supplementation with either an additional 1 μM enzyme + 1 mM ascorbic acid or fresh PASC substrate (0.275% w/v in suspension) + 1 mM ascorbic acid. Each reaction was performed in 50 mM bis-tris pH 6.8 and incubated at 37°C in a shaking incubator. Total oxidized ends were obtained by quantifying cellobionic acid by HPLC following hydrolysis of soluble products with *T. reesei* Celluclast enzyme cocktail. Each each timepoint represents the average of two independent assays measured singly by HPLC, with error bars indicating the standard error of the mean.


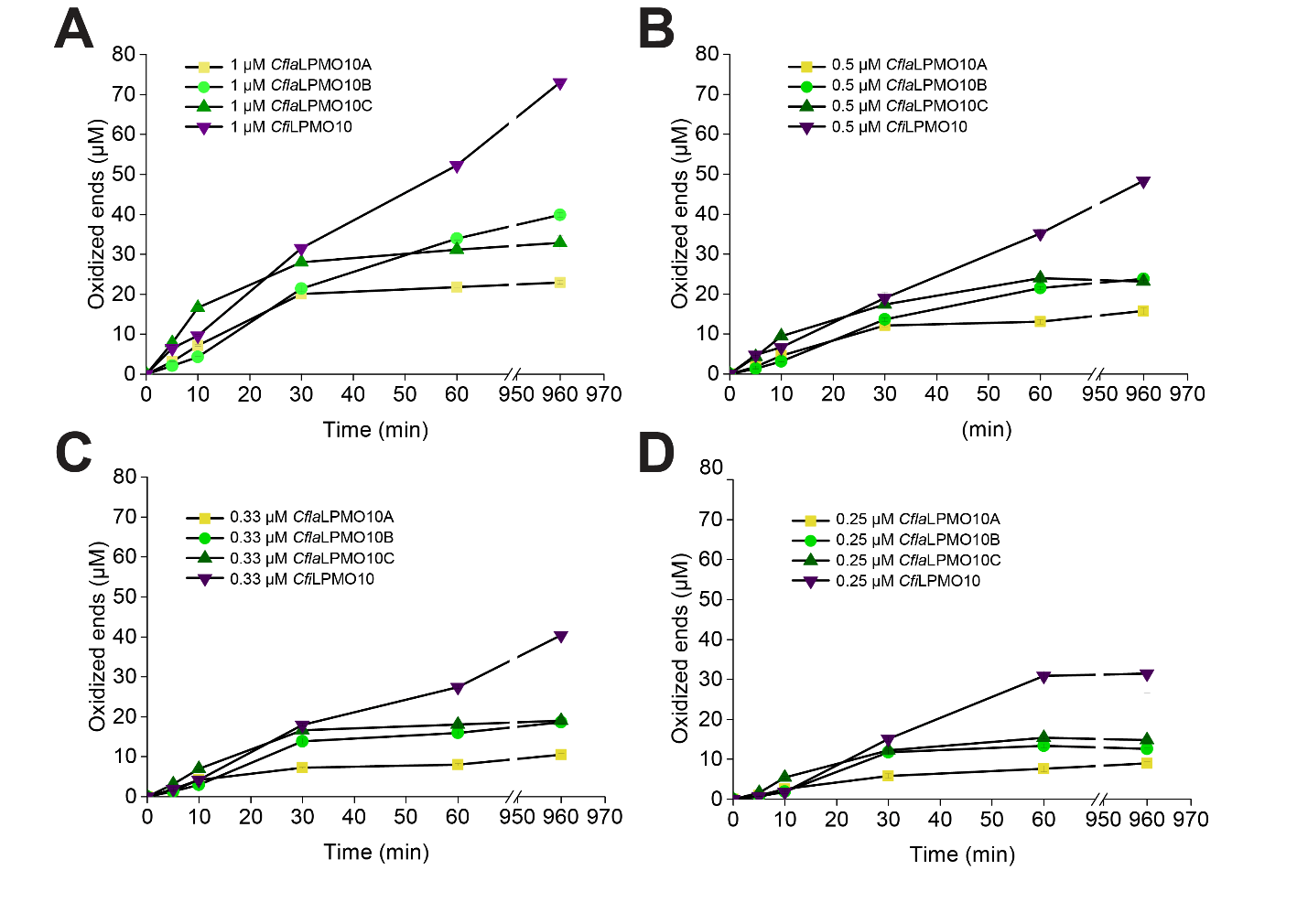


### Supplemental Figure S11. Effect of LPMO load on catalytic progress curve on PASC for all four *Cellulomonas* LPMOs. A-D: 1 - 0.25 μM total LPMO concentration reactions. Each reaction contained 0.1% substrate and 1 mM ascorbic acid and incubated at 37°C in a shaking incubator. Total oxidized ends were obtained by quantifying cellobionic acid by HPLC following hydrolysis of soluble products with *T. reesei* Celluclast enzyme cocktail. Each each timepoint represents the average of three independent assays measured singly by HPLC, with error bars indicating the standard error of the mean.


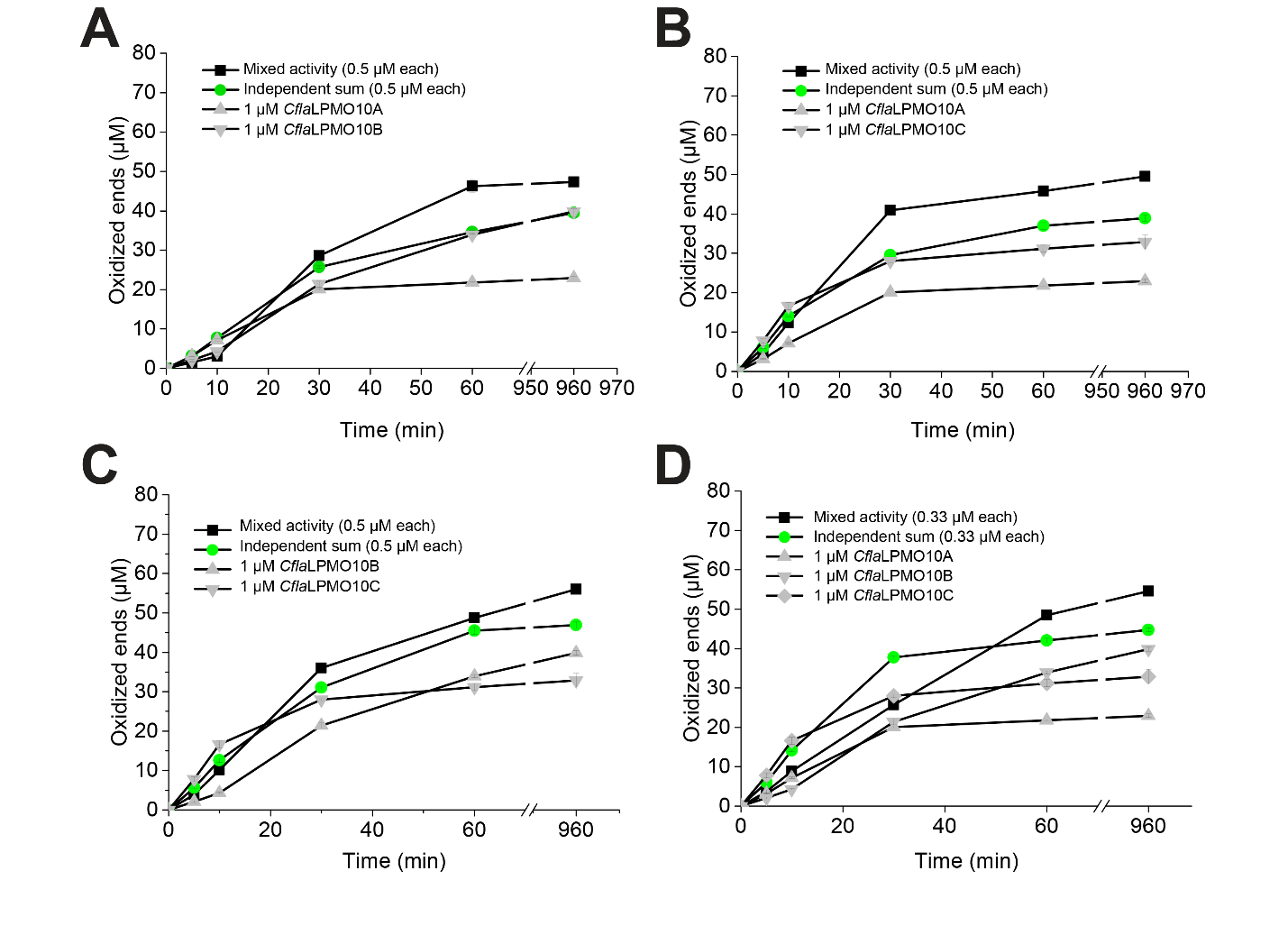


### Supplemental Figure S12. Progress curves of released oxidized products by *C. flavigena* LPMOs incubated in proportionate combinations of mixed and independent reactions. Mixed LPMO activity at double (A-C) and triple (D) combinations compared to the activity of individual LPMOs at the equivalent total enzyme loading. Each reaction contained 0.1% substrate and 1 mM ascorbic acid and incubated at 37°C in a shaking incubator. Total oxidized ends were obtained by quantifying cellobionic acid by HPLC following hydrolysis of soluble products with *T. reesei* Celluclast enzyme cocktail. Each each timepoint represents the average of three independent assays measured singly by HPLC, with error bars indicating the standard error of the mean.

**
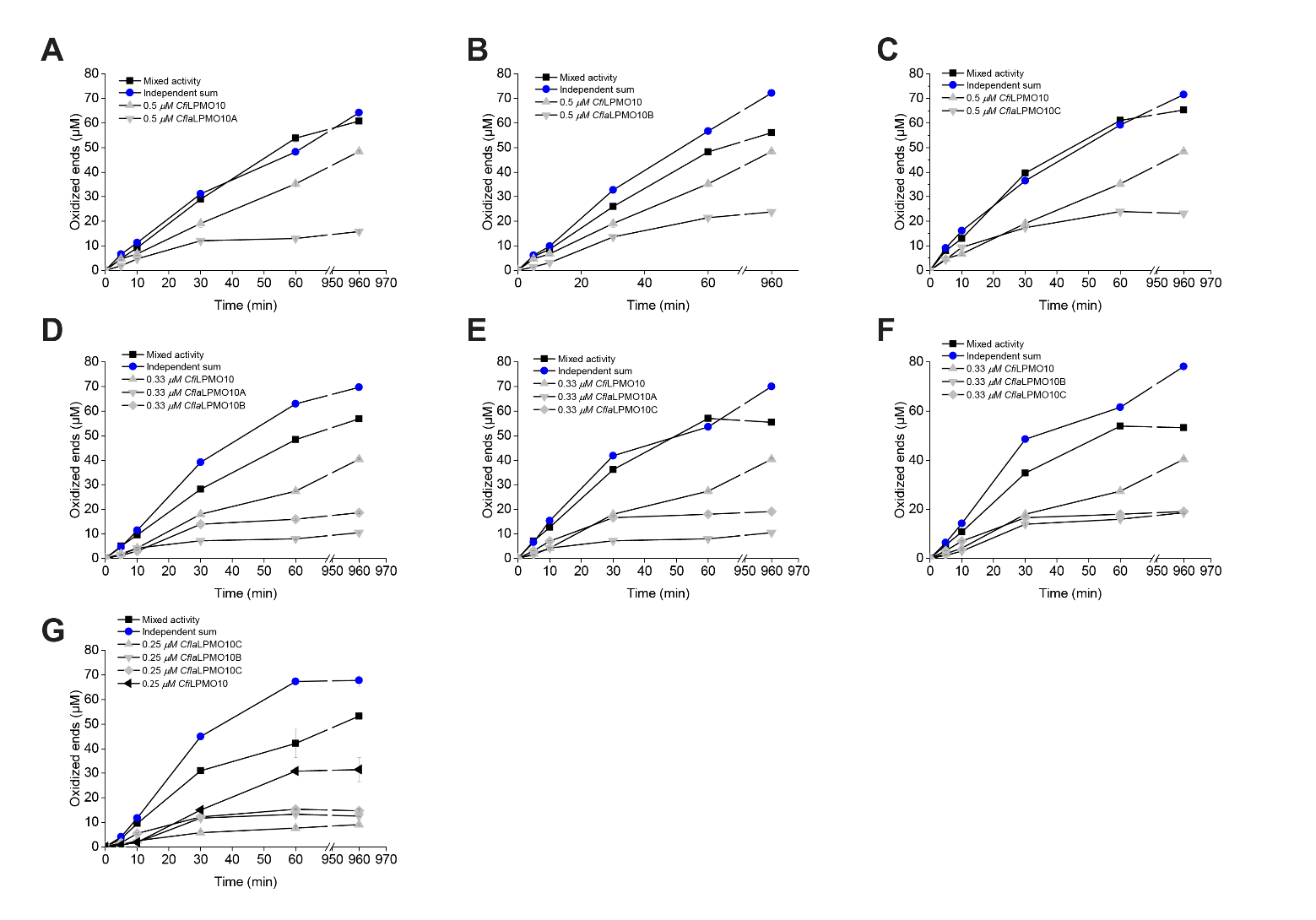
**

### Supplemental Figure S13. Progress curves of released soluble oxidized products by *C. fimi* LPMOs incubated in combination vs the sum total activity of independently assayed *C. flavigena* LPMOs. Each reaction contained 0.1% substrate and 1 mM ascorbic acid and incubated at 37°C in a shaking incubator. Total oxidized ends were obtained by quantifying cellobionic acid by HPLC following hydrolysis of soluble products with *T. reesei* Celluclast enzyme cocktail. Each each timepoint represents the average of three independent assays measured singly by HPLC, with error bars indicating the standard error of the mean.

## Supplemental Tables

### Supplemental Table 1. Percent identity (blue) and similarity (yellow) of *Cellulomonas* LPMO catalytic modules.

|  | *Cfla*LPMO10A  (*Cfla*_0175) | *Cfla*LPMO10B  (*Cfla_*0172) | *Cfla*LPMO10C  (*Cfla*_0316) | *Cfla*LPMO10D  (*Cfla*_0490) | *Cfi*LPMO10  (*Celf*_0270) |
| --- | --- | --- | --- | --- | --- |
| *Cfla*LPMO10A  (*Cfla*_0175) |  | 32 | 33 | 21 | 31 |
| *Cfla*LPMO10B  (*Cfla_*0172) | 50 |  | 60 | 19 | 74 |
| *Cfla*LPMO10C  (*Cfla*_0316) | 52 | 71 |  | 20 | 57 |
| *Cfla*LPMO10D  (*Cfla*_0490) | 31 | 29 | 30 |  | 27 |
| *Cfi*LPMO10  (*Celf*_0270) | 48 | 82 | 69 | 18 |  |

### Supplemental Table 2. Sum area of released C1-oxidized peaks (DP4-DP6) following refolding compared to non-boiled LPMO samples.

|  | *Cfla*LPMO10A | *Cfla*LPMO10B | *Cfla*LPMO10C | *Cfi*LPMO10 |
| --- | --- | --- | --- | --- |
| Control | 11 | 29 | 1.8 | 10.3 |
| After refolding | 12 | 23 | 1.3 | 8.5 |
| No enzyme | 0 | 0 | 0 | 0 |
